# The small GTPase RhoU lays downstream of JAK/STAT signaling and mediates cell migration in multiple myeloma

**DOI:** 10.1038/s41408-018-0053-z

**Published:** 2018-02-13

**Authors:** Sara Canovas Nunes, Martina Manzoni, Marco Pizzi, Elisa Mandato, Marilena Carrino, Laura Quotti Tubi, Renato Zambello, Fausto Adami, Andrea Visentin, Gregorio Barilà, Livio Trentin, Sabrina Manni, Antonino Neri, Gianpietro Semenzato, Francesco Piazza

**Affiliations:** 1 Department of Medicine, Division of Hematology, University of Padova, Padova, Italy; 2 Laboratory of Normal and Malignant Hematopoiesis, Venetian Institute of Molecular Medicine, Padova, Italy; 3 Hematology Unit, Fondazione IRCCS Ca’ Granda, Ospedale Maggiore Policlinico, Milan, Italy; 4 Department of Oncology and Hemato-Oncology, University of Milano, Milano, Italy; 5 Surgical Pathology and Cytopathology Unit, Department of Medicine - DIMED, University of Padova, Padova, Italy; 6 Present Address: Department of Medical Oncology, Dana-Farber Cancer Institute, Boston, MA USA

## Abstract

Multiple myeloma is a post-germinal center B-cell neoplasm, characterized by the proliferation of malignant bone marrow plasma cells, whose survival and proliferation is sustained by growth factors and cytokines present in the bone marrow microenvironment. Among them, IL-6 triggers the signal downstream of its receptor, leading to the activation of the JAK/STAT pathway. The atypical GTPase RhoU lays downstream of STAT3 transcription factor and could be responsible for mediating its effects on cytoskeleton dynamics. Here we demonstrate that *RHOU* is heterogeneously expressed in primary multiple myeloma cells and significantly modulated with disease progression. At the mRNA level, *RHOU* expression in myeloma patients correlated with the expression of *STAT3* and its targets *MIR21* and *SOCS3*. Also, IL-6 stimulation of human myeloma cell lines up-regulated *RHOU* through STAT3 activation. On the other hand, RhoU silencing led to a decrease in cell migration with the accumulation of actin stress fibers, together with a decrease in cyclin D2 expression and in cell cycle progression. Furthermore, we found that even though lenalidomide positively regulated RhoU expression leading to higher cell migration rates, it actually led to cell cycle arrest probably through a p21 dependent mechanism. Lenalidomide treatment in combination with RhoU silencing determined a loss of cytoskeletal organization inhibiting cell migration, and a further increase in the percentage of cells in a resting phase. These results unravel a role for RhoU not only in regulating the migratory features of malignant plasma cells, but also in controlling cell cycle progression.

## Introduction

Multiple myeloma (MM) is a post-Germinal Center cancer characterized by a multifocal proliferation of clonal, long-lived plasma cells (PCs) within the bone marrow (BM)^1^. This multistep malignancy is preceded by an age-progressive premalignant condition called monoclonal gammopathy of undetermined significance (MGUS)^1–3^. Some patients pass through a phase called smoldering myeloma (sMM), in which some of the diagnostic criteria for MM are met but there are no clinical manifestations^2^. In early stages, MM cells like normal long-lived PCs are highly dependent on the BM microenvironment that activates multiple pathways, protecting these cells from apoptosis^4^. IL-6, primarily produced by BM stromal cells (BMSCs), is the best characterized MM growth factor and is highly responsible for cell homing, seeding, proliferation, and survival through the activation of the JAK/STAT pathway^2,4^.

The Rho family of small guanosine triphosphatases (GTPases) forms part of the Ras super-family. These GTPases share a common biochemical mechanism, acting as molecular switches to transduce the signal downstream to their effectors^5^. To note, the Ras family has been proven to profoundly influence cell growth and activating mutations of Ras are associated with cancer^6^. In contrast, Rho GTPases are hardly ever found mutated but often display altered activity in malignant cells when compared to healthy counterparts^7^. Rho GTPases are potent regulators of cytoskeleton dynamics and of the actin filament system, thereby affecting the morphologic and migratory properties of cells^8^. Due to their important roles in controlling these cellular processes, deregulated Rho GTPases could be at the basis of many tumorigenic events.

The RhoU/V sub-family is particularly interesting due to its unique domain organization. Both members of this family, RhoU and RhoV, have an N-terminal proline-rich domain that is not present in any other Rho GTPase and that enables them to permanently bind to their effectors^7,9^. RhoU has no detectable GTPase activity but its very high intrinsic guanine nucleotide exchange activity is likely to ensure that the protein is predominantly in the GTP-loaded conformation^10^. It is encoded by the *RHOU* gene at 1q42.13 and its expression is mainly controlled at the RNA level downstream of Wnt-1 and STAT3 activation and it might mediate the effects of these signaling pathways in regulating cell morphology, cytoskeletal organization, and proliferation^11^. Also, different levels of this GTPase might lead to diverse outcomes in cell morphology. It is known that during epithelial-mesenchymal transition of neural crest cells, high levels of RhoU influence cell polarity and migration while low levels are required for cell adhesion^12^. While typical Rho proteins, such as Cdc42 and Rac1 that share significant sequence homology with RhoU, have an established role in cancer, very little is known about RhoU in tumorigenesis in particular in hematologic malignancies^7^. Since RhoU can alter cell adhesion, actin dynamics, and cell motility, we aimed at testing if this protein could mediate these cellular features in myeloma cells and if changes in its expression, and thus activity, might lead to BM niches remodeling.

## Materials and methods

### Patient samples and healthy donors

PCs were purified from BM samples using CD138 immunomagnetic microbeads (MidiMACS system, Miltenyi Biotec, Auburn, CA) and the purity of the positively selected PCs was >90% in all cases. Gene expression profiles (GEP) were investigated in a panel of 268 patients included in two different datasets and representative of all the major forms of PC dyscrasia: a proprietary dataset at NCBI Gene Expression Omnibus repository (accession #GSE66293) previously profiled by us (4 normal controls and 129 MM patients)^13,14^; and a publicly available data set including five normal controls, 20 MGUS, 33 SMM, and 41 MM patients (accession #GSE47552)^15^. The cohort consists of newly-diagnosed patients. The proprietary 129 MM tumors (accession #GSE66293) employed for the study were representative of the major molecular characteristics of the disease and stratified according the TC classification^16,17^. Deletions of 17p13, 13q14 and gain of 1q were also evaluated by FISH^18^. Procedures followed the rules indicated in the declaration of Helsinki. The internal Institutional Board approved the use of human material.

### Cell lines

INA-6 cell line was a kind gift from Dr. Martin Gramatzki (University of Kiel, Germany). U266, H929, RPMI-8226, and HS-5 were purchased from ATCC-LGC Standards (Milan, Italy). SaMMi cell line was generated in our laboratory^19^. Cell cultures were kept under controlled atmosphere at 37 °C in the presence of 5% CO_2_ and were periodically checked for Mycoplasma contamination.

### SiRNA transfection

U266 cells were transfected with Amaxa Nucleofector® Kit C (Lonza, USA) and 100 pmol of RhoU siRNA (Dharmacon ON-TARGETplus RHOU siRNA, Euroclone, Italy) or 100 pmol of Scrambled (Dharmacon ON-TARGETplus non-targeting siRNA, Euroclone, Italy) according to the manufacturer’s instruction.

### Chemicals

2 × 10^6^ cells were cultured in 2 ml of favorite medium, stimulated with 10 ng of IL-6 (ImmunoTools, Germany) and samples were collected at different time points. Cell lines that are usually cultured in medium supplemented with IL-6 were starved from this cytokine for 12 h previous to stimulus.

Stattic (Selleckchem, USA) was employed for 5 h at different doses to inhibit STAT3 and then IL-6 was added or not to the cells for 1 h. Cells were starved of IL-6 only during the 6 h of treatment.

Lenalidomide (Selleckchem, USA) was used at different doses for 4 or 24 h.

### Transwell migration assay

5μmTranswell® Permeable Supports on 24 well plates (Corning, USA) were used according to manufacturer instructions. Six hundred microliter non-supplemented medium + 0.1%BSA were added to the bottom of multi-well plate and 50 μL of the same mix were added on top of the transwell insert. 4 × 10^5^ cells were washed with HBSS buffer and then resuspended in 50 μL of recommended medium + 0.1%BSA and added to the top well insert. Plates were incubated for 20 min and then IL-6 stimulus (10 ng) or MSC culture medium (from 24 h MSC culture) was carefully added to the bottom well. IL-6 or MSC culture medium was not added to control wells. Plates were incubator for 6 h before collection.

### Immunohistochemistry (IHC)

IHC was performed on 4 μm-thick formalin-fixed, paraffin-embedded sections of BM biopsies of 1 healthy and 8 MGUS using anti-RhoU (HPA049592, Sigma-Aldrich, USA) and anti-IRF4 (HPA002038, Sigma-Aldrich, USA) monoclonal primary antibodies. Fifteen-miilimeter biopsies were stained with anti-RhoU and zones with a clearly abundant plasmacytosis were used in evaluating RhoU positivity. All sections were processed using the sensitive Bond Polymer Refine Detection kit, a biotin-free, polymeric horseradish peroxidase–linker antibody conjugate system, in an automated immunostainer (Bond maX, Menarini, Italy). Appropriate positive and negative controls were run concurrently. RhoU immunostain was semiquantitatively scored in a four-tiered scale, as follows: score 0 = negative staining; score 1 = weak positivity staining; score 2 = moderate positivity staining; score 3 = strong positive staining. Immunohistochemical reactions were independently scored by two investigators (agreement *k* > 0.8).

### Immunofluorescence (IF)

5 × 10^4^ cells were seeded on polylysine-coated glass slides and incubated at 37 °C for 1 h to let them adhere to the polylysine, fixed with formaldehyde 3.7%, permeabilized with Triton 0.1% and blocked with BSA 3%. Samples were then stained with Phalloidin Alexa Fluor 594 (Invitrogen, USA) for 30’, mounted in Vectashield mounting medium with DAPI (4′,6-diamidino-2-phenylindole) (Vector Laboratories, USA) and analyzed using ZEISS LSM700 confocal microscope with 63× magnification objective. Images were analyzed with ImageJ software.

## Results

### Rho GTPases display altered expression in MM PCs

**Fig. 1:**
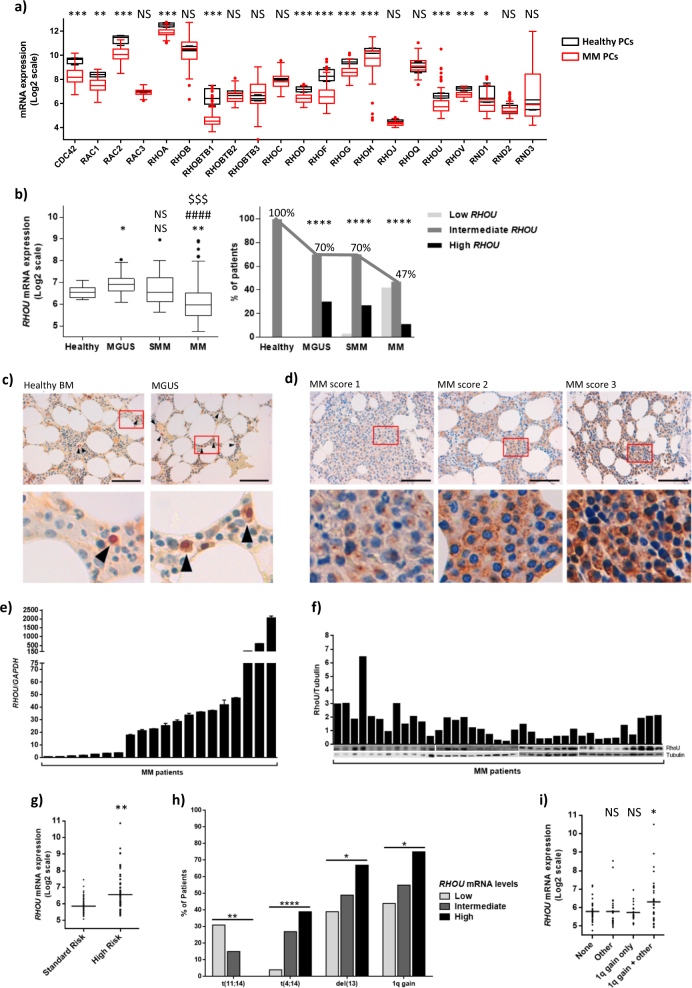
RhoU is heterogeneously expressed in MM. **a** Rho GTPase family members’ expression in healthy and MM PCs. Box plot showing mean ± SD of healthy PCs from four donors (red) and mean ± SD of MM PCs from 129 MM patients at diagnosis (black). In the x axis are all the members of the Rho GTPase family in alphabetical order. Student’s *t* test *p < 0.05; **p < 0.01; ***p < 0.001 when compared to healthy PCs. **b** left: *RHOU* expression in the different steps of MM progression. Box plot showing mean ± SD of healthy PCs from nine donors, MGUS PCs from 20 patients, sMM PCs from 33 patients and MM PCs from 170 patients at diagnosis. Student’s *t* test: NS non significant; *p < 0.05; **p < 0.01; ***p < 0.001; ****p < 0.0001 (*compared to healthy; ^#^compared to MGUS; ^$^compared to SMM). right: Bar chart showing the percentage of patients for each step of the disease that fall in the diverse groups of *RHOU* expression. Patients were divided in three groups with low (<mean−2 SD of healthy controls), intermediate, and high (>mean + 2 SD of healthy controls) *RHOU* expression. Chi square test of patients’ distribution: ****p < 0.0001 when compared to healthy. **c** RhoU expression in BM biopsies of a healthy donor and a representative MGUS patient. PCs in the samples are indicated with black arrows. Nucleuses of PCs are positive for IRF4 staining and are therefore red. **d** IHC staining showing low (MM score 1), intermediate (MM score 2) and high (MM score 3) positivity for RhoU staining in BM biopsies from representative MM patients. **c** and **d** Original magnification x60; inset with x5 digital zoom; scale bar = 100 μm. **e** Bar charts showing qRT-PCR RHOU/GAPDH ratio values in PCs purified from 20 MM patient’s biopsies. Mean ± SD of technical triplicates is shown for each samples. **f** Immunoblot and densitometry of RhoU/Tubulin in PC lysates from 39 MM patient’s biopsies. **g** Aligned dot plot with *RHOU* expression in patients that fall in the standard risk (TC1 and TC2) and high risk (TC3, TC4, and TC5) groups. Mean expression in each group is marked with a bar. *p* value was calculated by Student’s *t* test. **h** Bar chart representing the percentage of patients from each of the three groups with low (<mean—2 SD of healthy controls), intermediate, and high (>mean + 2 SD of healthy controls) *RHOU* expression that exhibit a particular genetic alteration. Student’s *t* test for trend *p < 0.05; **p < 0.01; ****p < 0.0001. **i** Aligned dot plot with *RHOU* expression in patients that don’t present any unfavorable cytogenetic alterations (none), that present unfavorable cytogenetic alterations but not 1q gain (other), that present only 1q gain, and that present 1q gain combined with other unfavorable cytogenetic alterations. Student’s *t* test: NS non significant; *p < 0.05

We have assessed the expression of the Rho GTPase family members in PCs from BM biopsies of MM patients and in normal BM PCs from healthy donors, using the gene expression profiling (GEP) of 129 MM patient samples at diagnosis and four healthy controls included in the proprietary GEO data set GSE66293. We found that Rho GTPase family members are differently expressed in MM PCs when compared to healthy PCs (Fig. 1a). More precisely, 12 (*CDC42, RAC1, RAC2, RHOA, RHOBTB1, RHOD, RHOF, RHOG, RHOH, RHOU, RHOV,* and *RND1)* out of the 21 GTPases analyzed, were expressed at significantly lower levels in MM when compared to healthy controls (*p* < 0.05).

### RhoU is heterogeneously expressed in different steps of MM progression

Next, we combined the data available in two GEP data sets (#GSE66293 and #GSE47552) as previously described^13^ and found that *RHOU* expression is significantly and negatively modulated with disease progression, being over-expressed in BM PCs from MGUS patients and down-modulated in BM PCs from most MM patients (Fig. 1b left panel). However, we found a large heterogeneity in *RHOU* expression, with several patients showing high transcript levels even in late stages. Therefore, we divided patients in three groups according to low (<mean—2 SD of healthy controls), intermediate (=mean ± 2 SD of healthy controls), and high (>mean + 2 SD of healthy controls) expression. Along with disease progression there was an evident decrease in the percentage of patients that fell in the group with intermediate *RHOU* expression (Fig. 1b right panel) and an increase in heterogeneity. In MM in particular, there were three distinct groups of patients: 42% with low, 47% with intermediate, and 11% with high *RHOU* expression.

To further examine RhoU expression we performed IHC analysis in an independent panel of patients including 8 MGUS and 15 active MM. All MGUS patients’ PCs showed a high intensity cytoplasmic staining for RhoU when compared to healthy PCs (Fig. 1c). Also, PCs from all MM patients showed positivity for RhoU staining and were given a score based on the intensity. More precisely 20% showed weak (score 1), 40% moderate (score 2), and 40% high RhoU (score 3) positivity, consistent with the heterogeneous levels of *RHOU* mRNA described above (Fig. 1d). Cells with different scores seem to also have a diverse localization of RhoU: in MM cells with score 1 RhoU had a dot-like localization in proximity of the nucleus, which could represent the actin microtubule-organizing center, whereas cells that scored 2 or 3 showed a granular cytoplasmic and membrane localization. The heterogeneous levels of RhoU in MM patients were also validated by qRT-PCR (Fig. 1e) and Western Blot (Fig. 1f).

Next, we evaluated *RHOU* levels according to the major molecular groups of MM patients based on the TC classification as previously described^17^. Patients were further divided in two groups: standard risk patients that fall into TC1 and TC2 groups (*n* = 64) and characterized by *t*(11,14) (TC1) and hyperdiploidy (TC2) leading to cyclin D1 expression, and high risk patients that comprise TC3, TC4, and TC5 groups (*n* = 65) characterized by cyclin D2 expression alone (TC3) and presence of *t*(4;14) (TC4) or MAF translocations (TC5). The results show that patients in the high risk group have significantly higher levels of *RHOU* expression (Fig. 1g), compared to TC1/TC2 patients. Furthermore, patients with high *RHOU* expression have a higher frequency of 1q gain, del(13), and t(4;14) (Fig. 1h). Finally, since *RHOU* is located at 1q42 we have divided the patients in four groups, without unfavorable cytogenetic alterations (none), with unfavorable cytogenetic alterations but without 1q gain (other), with 1q gain only, and with 1q gain plus unfavorable cytogenetic alterations, discovering that 1q gain alone is not enough to cause a significant increase in *RHOU* expression (Fig. 1i).

To better understand if *RHOU* expression could correlate with patient survival we have used two datasets: one with expression in untreated patients and overall survival after Total Therapy 2 (TT2) and Total Therapy 3 (TT3) regimens (#GSE2658)^20–22^, and another with expression after several lines of therapy and overall survival after Total Therapy 6 (TT6) (#GSE57317)^23^. We have divided the patients from each dataset in *RHOU* expression quartiles and compared the survival curves of the first and fourth quartiles. In TT2 or TT3 regimens there is a 50% higher risk of death (hazard ratio = 1.50) for the patients in the 4th quartile when compared to patients in the I quartile (Supplementary Figure S1a and b). In TT6 regimen the risk of death increases to 612% (hazard ratio = 7.12) for patients in the IV quartile when compared to patients in the I quartile (Supplementary Figure S1c and d). These data suggest a positive correlation between high RHOU levels and worse prognosis also after anti-myeloma therapies. Whether or not its association with poor outcomes can be linked to the combination of 1q gain and other unfavorable cytogenetic alterations remains to be investigated.

### Patients with high and low *RHOU* expression have a different GEP

**Fig. 2:**
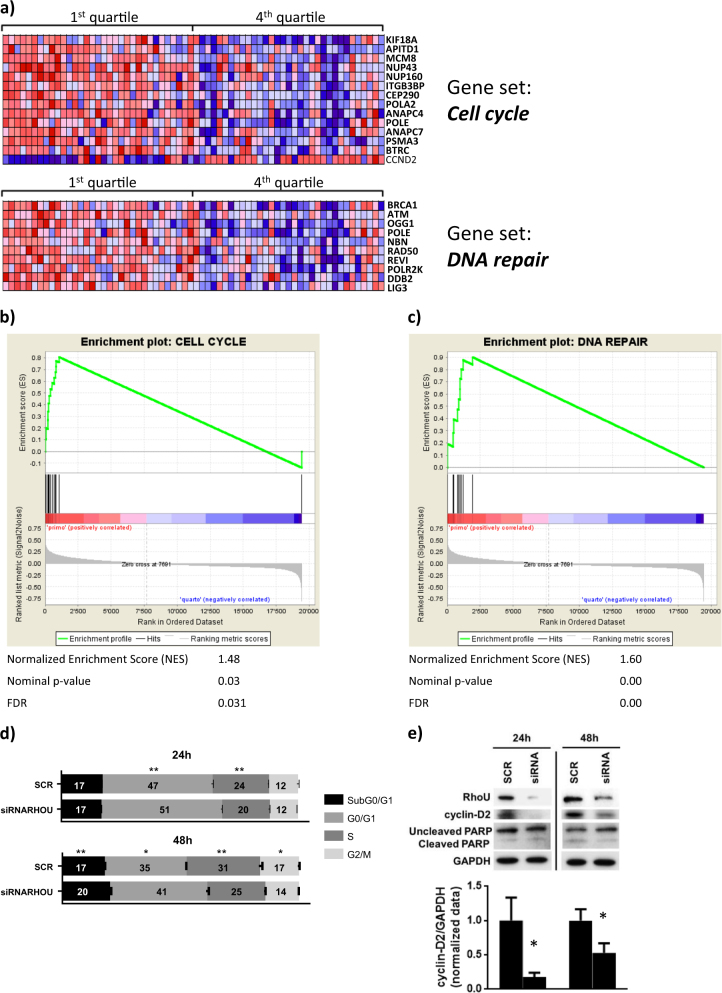
Patients with high and low *RHOU* expression have different gene expression profiles. **a** Heat map of genes contributing to the core enrichment in the “Cell Cycle” and “DNA Repair” gene sets detected by GSEA analyzing significantly up-regulated genes (*p*-value ≤ 0.05) in *RHOU* quartile I versus IV (indicated in bold). Enrichment plots for **b** “Cell Cycle” and **c** “DNA Repair” gene sets. The green curves show the enrichment score and reflect the degree to which each gene (black vertical lines) is represented at the bottom of the ranked gene list. **d** Cell cycle analysis based on PI staining of SCR and siRNA RHOU cells 24 and 48 h after transfection. Data represents mean ± SD of three independent experiments. Student’s *t* test: *p < 0.05; **p < 0.01. **e** Representative immunoblots from six samples from two independent experiments of RhoU and cyclin D2 expression 24 and 48 h after siRNA transfection, and densitometry of the mean expression of cyclin D2/GAPDH ± SD

To gain insights into the role of *RHOU* in MM, we focused on our proprietary MM group (#GSE66293) due to the absence of any bias related to sample molecular characteristics, and looked for the gene expression signature associated with *RHOU* expression. Specifically, we ranked patients in four classes based on *RHOU* expression level. Next, by comparing the lowest (I quartile) with the highest (IV quartile) *RHOU* expressing patients, we found 557 genes differentially expressed between the two groups that appeared to be gradually modulated in the four quartiles (Supplementary Table S1). Functional enrichment analysis of the 557 deregulated genes using DAVID Bioinformatics Resources 6.8 evidenced 12 significantly annotation clusters (ES > 1.3) and six significantly enriched pathways, among which cell cycle and DNA repair (Supplementary Table S2). In addition, to confirm the correlation between *RHOU* and these pathways, we used the Gene Set Enrichment Analysis (GSEA) to identify a priori defined sets of genes showing concordant modulation between patients with low and high *RHOU* expression level. Notably, the cell cycle control and DNA repair gene sets were found highly enriched in MM group expressing lower (I quartile) *RHOU* levels (Fig. 2a-c).

*RHOU* clustered with 14 genes that regulate the cell cycle and the mitotic process (Table 1). *CCND2* was the only gene in the list that positively correlated with *RHOU* (Pearson’s *r* = 0.402; *p*-value = 7.193e-15) suggesting that malignant PCs with high *RHOU* expression have increased levels of cyclin D2 and a higher replication rate. All the other genes responsible for cell cycle control and DNA damage response had a negative correlation with *RHOU* expression (Fig. 2b). *RHOU* also clustered with 10 DNA damage repair genes in the “DNA Repair” pathway (Table 2). All the ten genes in the list negatively correlate with *RHOU* expression (Fig. 2c).

**Table 1:** Gene set “Cell Cycle” was detected by GSEA as significantly up-regulated in RHOU quartile I versus IV

GSEA results summary	
GeneSet	Cell cycle
Genes	ITGB3BP, NUP160, BTRC, POLE, ANAPC4, KIF18A, POLA2, MCM8, APITD1, CCND2, PSMA3, CEP290, ANAPC7, NUP43
Normalized Enrichment Score (NES)	1.48
Nominal *p*-value	0.03
FDR	0.031

ITGB3BP overexpression induces apoptosis in cancer cells^24^; NUP160, NUP43, KIF18A, BTRC, APITD1, and CEP290 are required for correct mitosis, centrosome dynamics, and chromosome alignment^25–30^; POLE, POLA2, and MCM8 have extremely important roles in DNA replication and genome stability^31–33^; ANAPC4 and ANAPC7 have emerging roles in differentiation control, genomic stability and tumor suppression^34^; CCND2 enhances cell cycle progression and MM proliferation^35^ and PSMA3 is part of the proteasome complex and essential for protein degradation during cell cycle progression^36^.

**Table 2:** Gene set “DNA Repair” was detected by GSEA as significantly up-regulated in RHOU quartile I versus IV

GSEA results summary	
GeneSet	DNA repair
Genes	NBN, REV1, POLR2K, POLE, DDB2, LIG3, OGG1, ATM, RAD50, BRCA1
Normalized Enrichment Score (NES)	1.60
Nominal *p*-value	0.00
FDR	0.00

These genes are extremely important since they encode for proteins essential in a number of cellular pathways that maintain genomic stability, including DNA damage-induced cell cycle checkpoint activation, DNA damage repair, protein ubiquitination, chromatin remodeling, as well as transcriptional regulation and apoptosis^37–44^.

Strikingly, in U266 cell line, the inhibition of RhoU by siRNA resulted at 24 h in a slight but significant accumulation of cells in the G0/G1 phase and a decrease in replicating S phase cells (Fig. 2d). The results were more evident at 48 h with an increase in both sub G0/G1 and G0/G1 phases and a decrease in both S and G2/M phases. Since G1 to S phase transition is tightly regulated by the expression of cyclin D2, we have analyzed this protein’s expression after RhoU silencing and confirmed a decrease in its levels as early as 24 h after transfection; The decrease in RhoU protein levels at these time points was confirmed by western blotting (Fig. 2e).

### *RHOU* is overexpressed in IL-6-dependent MM cell lines and is driven by the activation of STAT3

**Fig. 3:**
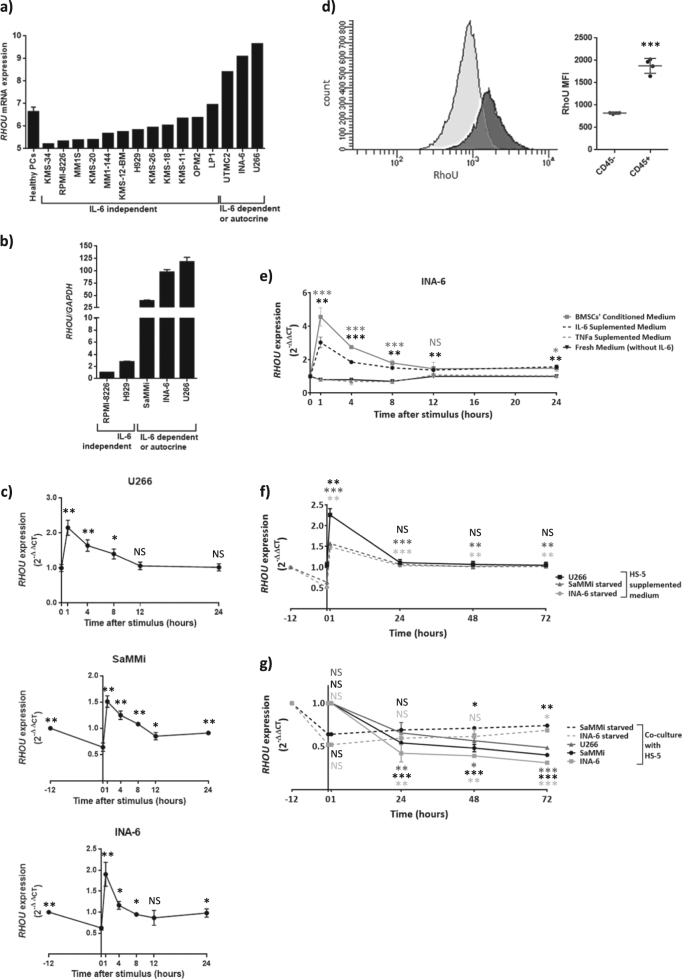
*RHOU* expression in MM cell lines relies on IL-6 stimulus. **a** Bar chart showing *RHOU* expression assessed by GEP (#GSE66293) in MM cell lines as compared to healthy PCs. **b** Bar chart showing *RHOU* expression in MM cell lines assessed by RT-PCR. Data represents normalized mean ± SD of three independent samples of each cell line. **c** Time-course of *RHOU* expression after IL-6 stimulus showing a significant increase in *RHOU* expression as early as one hour after stimulus; time points before zero correspond to IL-6 starvation period. **d** Left: representative dot plot showing intracytoplasmic RhoU staining in the CD45+ and CD45− populations of U266 cell line. Right: mean ± SD of four samples mean fluorescence intensity of intracytoplasmic RhoU staining in the CD45+ and CD45− populations of U266 cell line. **e** Time-course of *RHOU* expression after the addition of IL-6, TNF-α, BMSCs’ conditioned medium or fresh unsupplemented medium. f Time-course showing the changes in *RHOU* expression after the addition of HS-5 supplemented medium of starved and not starved cell lines. **g** Time-course showing the changes in *RHOU* expression after co-culture with HS-5 of starved and not starved cell lines. **c**, **e**, **f**, and **g** Data represents mean ± SD of six samples from at least two independent experiments normalized over mean expression at time zero. Student’s *t* test: NS non significant; *p < 0.05; **p < 0.01; ***p < 0.001, when compared to time zero of untreated

To better understand *RHOU* regulation in MM we have investigated its expression on the GEP data (#GSE66293) and on five MM cell lines available in our laboratory, two IL-6 dependent (INA-6 and SaMMi), one that autocrinally produces IL-6 (U266) and two IL-6 independent (H929 and RPMI-8226). GEP data relative to MM cell lines shows that only INA-6, U266 and UTCM2 (a cell line that also autocrinally produces IL-6) had high *RHOU* expression levels (Fig. 3a). We have also investigated *RHOU* expression by qRT-PCR confirming that RPMI-8226 and H929 expressed *RHOU* at very low levels, while IL-6 dependent/autocrine cell lines over-expressed it (Fig. 3b).

To determine whether IL-6 stimulus could lead to an up-regulation of *RHOU* expression cells were deprived from this cytokine and subsequently stimulated. Samples were collected at different time points (Fig. 3c). All three IL-6-dependent cell lines up-regulated *RHOU* as early as 1 h after stimulus. In detail, 1 h after the addition of IL-6, there was an increase in *RHOU* expression equal to 1.2 times-fold in U266, 1.7 times-fold in SaMMi, and 2.4 times-fold in INA-6. U266 might display the smallest changes due to the fact that this cell line cannot be starved from IL-6 since it autocrinally produces it.

Since CD45^+^ myeloma cells have increased JAK/STAT activation^45,46,^, we have also studied the CD45^+^ and CD45^−^ U266 cell populations to further examine RhoU expression in this context. Intracytoplasmic RhoU staining showed a mean fluorescence intensity (MFI) of 822 and of 1878 in the CD45^−^ and CD45^+^ cell populations, respectively (Fig. 3d).

To verify if this effect was due to the specific activation of the IL6R/STAT3 pathway, we stimulated cells also with TNF-α, conditioned medium from BMSCs’ culture, or unsupplemented fresh medium (Fig. 3e). The dynamics of *RHOU* expression after the addition of BMSCs’ conditioned medium or IL-6 stimulus were similar, while TNF-α did not cause any significant changes. Adding derived media from HS-5 stromal cell line showed the same results as IL-6 stimulus (Fig. 3f) and a really slow increase in *RHOU* expression was obtained by co-culturing IL-6 starved cell lines with HS-5 cells (Fig. 3g). However, the co-culture on HS-5 cells of non-starved cells, with high levels of RhoU, resulted in an actual decrease in *RHOU* expression (Fig. 3g). These results may support the hypothesis of a contact dependent down-modulation of *RHOU* after cell adhesion, as previously observed in neural crest cells^47^.

**Fig. 4:**
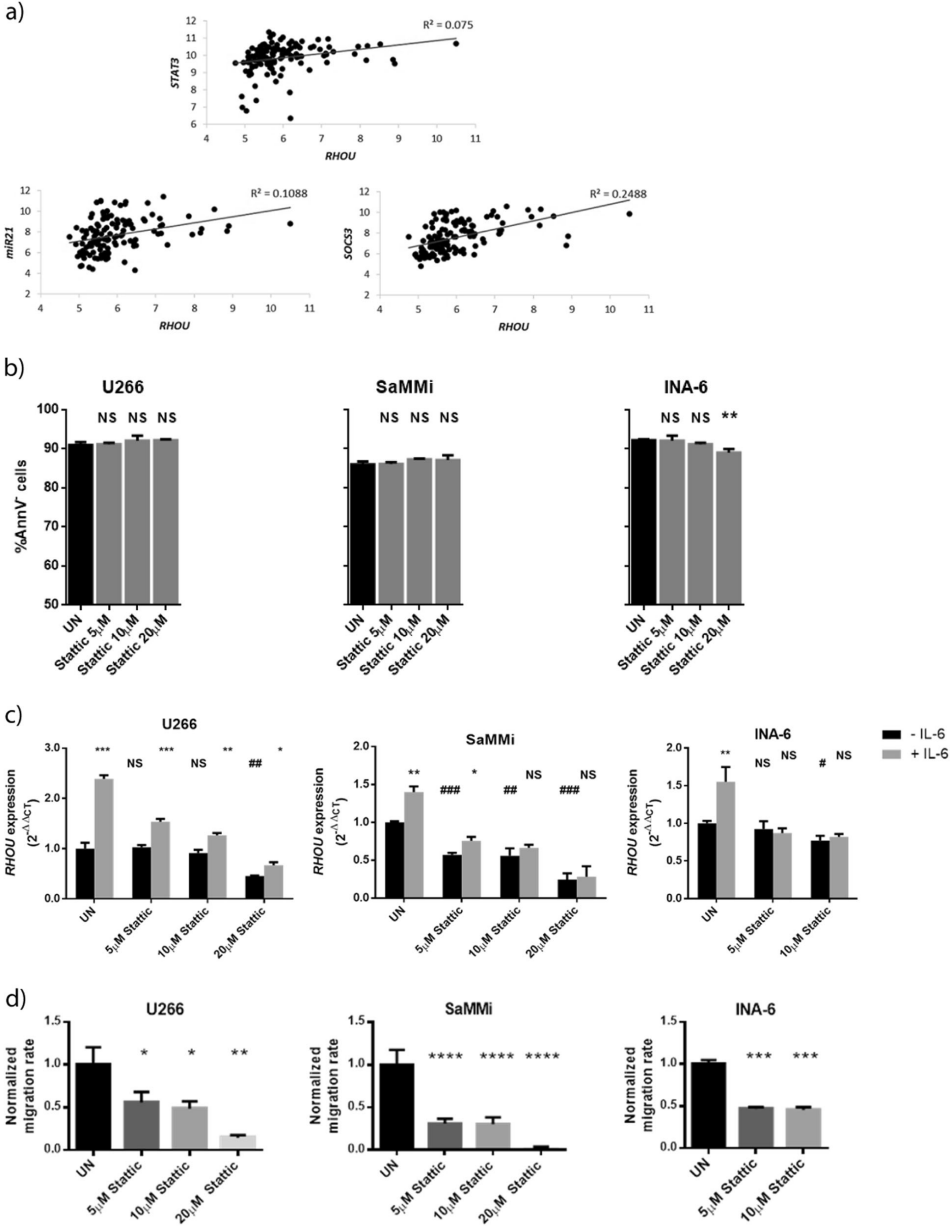
In MM cell lines *RHOU* expression lays downstream of STAT3 activation. **a** Correlation between* RHOU* expression and *STAT3* expression (Pearson’s *r* = 0.2739; *p*-value = 0.0017) or *MIR21* expression (Pearson’s *r* = 0.3299; *p*-value = 0.0001) or *SOCS3* expression (Pearson’s *r* = 0.4988; *p*-value = 1.786e-09). **b** Bar charts showing the percentage of live cells (AnnV^−^) after treatment with different doses of Stattic. **c** Bar charts showing *RHOU* expression after STAT3 inhibition before and after IL-6 stimulus. Black bars represent *RHOU* expression after 6 h of culture with different concentrations of stattic. Grey bars show *RHOU* expression after 5 h of stattic treatment + 1 h of combined treatment with IL-6 stimulus. *Compared with same treatment conditions without IL-6 stimulus, ^#^Compared to UN. **d** Bar charts exhibiting normalized mean cell count ± SD of four samples from at least two independent experiments, after treatment with increasing concentrations of stattic, accessed by migration assay with IL-6, normalized over mean migration of UN. **b**, **c**, and **d** UN untreated; student’s *t* test: NS non significant; *p < 0.05; **p < 0.01; ***p < 0.001; ****p < 0.0001

Interestingly, looking at GEP data from MM patients we found a weak but significant positive correlation between *RHOU* and *STAT3* expression. The analysis of STAT3 targets *MIR21* and *SOCS3* unraveled however a stronger positive correlation with the expression of *RHOU* (Fig. 4a).

Next, to verify that STAT3 was the principal transcriptional regulator of *RHOU* downstream of the IL-6R, we inhibited STAT3 with stattic, a chemical inhibitor that blocks STAT3 phosphorylation, dimerization, and nuclear transition by specifically binding to its SH2 domain^48^. Based on annexin V staining, a 6 h treatment with this drug resulted in a slight but significant increase in cell death only at the highest doses for INA-6 cell line (Fig. 4b). Upon non-toxic doses of stattic treatment we observed a dose-dependent decrease in *RHOU* expression, while cells were less, if not able, to upregulate the GTPase in response to IL-6 stimulus (Fig. 4c). Stattic treatment also led to a dose dependent decrease in cell migration that could rely on *RHOU* expression (Fig. 4d).

### Lenalidomide treatment increases MM cell migration that is overturned by RhoU inhibition

**Fig. 5:**
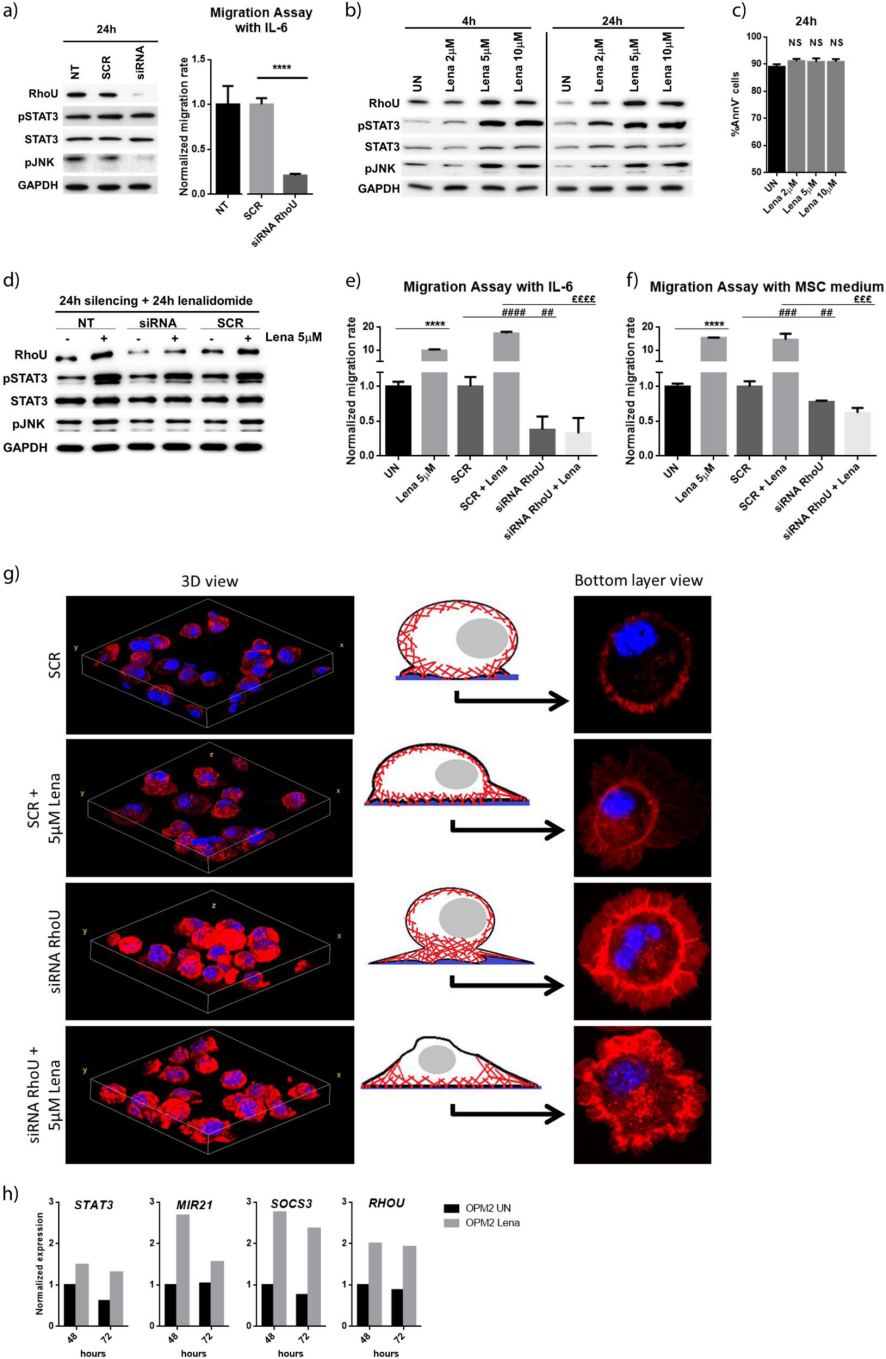
Lenalidomide enhances MM cell migration through the upregulation of RhoU. **a** Immunoblot of U266 proteins and bar chart exhibiting normalized mean cell count ± SD of four samples from at least two independent experiments, after 24 h of transfection with SCR or RhoU siRNA. **b** Immunoblots of U266 proteins after 4 and 24 h of treatment with different doses of lenalidomide. **c** Bar charts showing the percentage of live cells (AnnV^−^) after treatment with different doses of lenalidomide for 24 h. **d** Immunoblot of U266 proteins after 48 h of transfection with RhoU or SCR siRNA, lenalidomide was added in the last 24 h. **a**,** b**, and **d** show representative immunoblots from six samples from at least two independent experiments; UN untreated, NT not transfected; protein expression is showed in the following order: RhoU, phospho-STAT3(Tyr705), STAT3, phospho-JNK(Thr183/Tyr185), and GAPDH. **e** Bar charts showing normalized mean cell count ± SD of four samples from at least two independent experiments after 48 h of transfection with RhoU or SCR siRNA combined with lenalidomide treatment in the last 24 h accessed by migration assay with IL-6, normalized over mean migration of UN. **f** Bar charts showing normalized mean cell count ± SD of four samples from at least two independent experiments after 48 h of transfection with RhoU or SCR siRNA combined with lenalidomide treatment in the last 24 h accessed by migration assay with MSC medium, normalized over mean migration of UN. **e** and **f** UN untreated *Compared to UN; ^#^compared to SCR; ^**£**^compared to SCR + Lena, *p < 0.05; **p < 0.01; ***p < 0.001; ****p < 0.0001. **g** Changes in the actin cytoskeleton after RhoU silencing or SCR transfection combined or not with lenalidomide treatment. DAPI staining shows the nucleus in blue, phalloidin staining in red shows F-actin filaments. Cells were fixed after 48 h siRNA transfection and 24 h of 5 μM lenalidomide treatment. All cells were plated at the same concentration in a six well plate for 48 h to maintain growth conditions. **h** Expression of STAT3 and its targets MIR21, SOCS3, and RHOU after treatment of OPM2 cell line with lenalidomide for 48 and 72 h (#GSE31421)

To better assess the effects of RhoU in MM cells we have silenced it by transfecting cells with specific siRNA particles. Scrambled non-targeting siRNA (SCR) was used as control. RhoU silencing led to a clear decrease in JNK activating phosphorylation, but did not seem to have an effect on cell death nor on STAT3 activation (Fig. 5a left panel). JNK1 is a target of RhoU, important for the development of filopodia^11^. Therefore, an impairment in its activation could explain the decreased migration capability of RhoU-targeting siRNA transfected cells (Fig. 5a right panel). Furthermore, since IMIDs were shown to be able to regulate the activation of classical Rho proteins in human monocytes^49^, we aimed at studying the effects of lenalidomide on RhoU expression. Immunoblot analysis showed an increase in STAT3 activation after treatment with lenalidomide (Fig. 5b) without a clear effect in viability in U266 (Fig. 5c) and in INA-6 (Supplementary Figure S2a) cell lines. We also observed an increase in RhoU expression and in JNK activating phosphorylation (Fig. 5b and Supplementary Figure S2a) that could result in increased cell mobility. When combining RhoU inhibition with lenalidomide we observed that JNK phosphorylation remained unchanged instead of increasing as happened in UN and SCR conditions (Fig. 5d). When comparing siRNA and SCR transfected cells we observed a significant decrease in migration (Fig. 5e and f). RhoU is thus essential for JNK activation and MM cell migration. Treatment with lenalidomide, that consequently determined higher RhoU and active JNK levels, resulted in a boost in cell migration (Fig. 5e and f; and Supplementary Figure S2b and c). RhoU silencing abolished lenalidomide induced migration (Fig. 5e and f).

We further studied cytoskeleton changes and alterations in cell adhesion by employing IF techniques. Although all cells were able to adhere to polylysine coated glass, the changes in cell morphology were evident. SCR cells displayed a normal round-up morphology (3D view) with smooth edges and few spiky protrusions (bottom layer view); siRNA RhoU cells had bigger lamellipodia-like edges with an accumulation of stress fibers and focal adhesions; lenalidomide treated SCR cells were flattened (3D view) and with multiple filopodial protrusion (bottom layer view), typical of migrating cells in accordance with the migration data above; noteworthy, the combination of RhoU silencing with lenalidomide treatment resulted in a loss of cytoskeleton organization (Fig. 5g).

To deepen the study of STAT3 activation and RhoU overexpression upon lenalidomide treatment, we have analyzed the expression of STAT3 itself and its target genes MIR21, SOCS3 and RHOU in a GEP dataset of OPM2 cell line upon lenalidomide treatment for 48 and 72 h (#GSE31421)^50^. Upon lenalidomide treatment there is a 50% increase in STAT3 expression, while the increase in the expression of its target genes is even more clear (Fig. 5h).

**Fig. 6:**
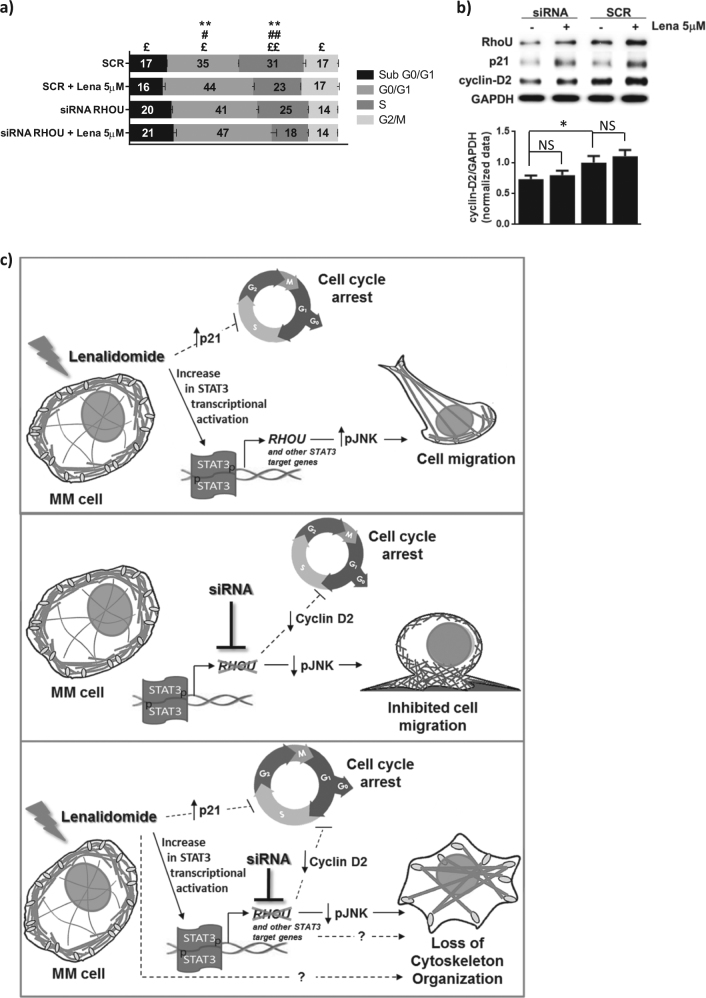
Cell cycle analysis and schematic view of the effect of lenalidomide, RhoU silencing or their combination in MM cells. **a** Cell cycle analysis based on PI staining of SCR and siRNA RHOU cells 48 h after transfection with the addition of lenalidomide in the last 24 h. Data represents mean ± SD of three independent experiments. Student’s *t* test: *SCR compared to SCR + Lena; ^#^siRNA RhoU compared to siRNA RhoU + Lena; ^£^siRNA RhoU + Lena compared to SCR + Lena, *p < 0.05; **p < 0.01. **b** Representative immunoblots from six samples from at least two independent experiments of RhoU, cyclin D2, and p21 expression after siRNA transfection and lenalidomide treatments, and densitometry of the expression of cyclin D2/GAPDH. **c** Lenalidomide treatment determines an increase in STAT3 activation and consequently the increased expression of STAT3 target genes including RhoU. This, combined with increased levels of pJNK, results in a reorganization of the cytoskeleton and boosts cell migration. These effects are parallel to cell cycle arrest, through a mechanism that is not explored in this manuscript. RhoU inhibition, on the other hand, results in decreased levels of active JNK leading to decreased cell migration rates and increased lamellipodial protrusions. RhoU silencing also caused MM cell cycle arrest due to a decrease in the expression of cyclin D2. The combination of both RhoU silencing and lenalidomide treatment further inhibits cell cycle progression and overcomes lenalidomide induced migration by decreasing the levels of active JNK causing however a loss of cytoskeleton organization. This cytoskeleton organization loss may be due to the expression of other STAT3 target genes or due to lenalidomide targets not explored in this article

Additionally, since RhoU silencing caused cell cycle arrest we further investigated the changes in cell cycle after lenalidomide treatment or the combination of both. Twenty four-hour treatment with lenalidomide caused a significant increase in G0/G1 phase and a decrease in S phase (Fig. 6a). The combination of both RhoU silencing and lenalidomide led to a further accumulation of cells in the G0/G1 phase (Fig. 6a). We have also analyzed cyclin D2 expression, unraveling that although lenalidomide treatment impaired the transition from G1 to S phases, it did have a significant impact in cyclin D2 expression (Fig. 6b). However, as previously described in literature, lenalidomide treatment caused an increase in p21 expression^51^ in both conditions that might explain the impairment in cell cycle progression.

A schematic view of the proposed mechanism is depicted in Fig. 6c.

## Discussion

We report here the first evidence of a clear unbalance in the expression of multiple members of the Rho GTPase family in MM PCs when compared to normal PCs. Interestingly, opposite to what was seen in other tumors where there is an over-expression of some of the members of this family^7^, we have found that more than 50% of Rho GTPases are actually down-modulated in MM PCs when compared to healthy PCs.

Focusing our attention on the RhoU/V family, we have found that RhoU expression is significantly modulated during the different steps of MM progression. MGUS patients showed higher RhoU levels when compared to normal controls, raising the hypothesis that this protein might be important especially in the early stages of MM malignancy. With disease progression and accumulation of malignant PCs in the BM, we observed a decrease in *RHOU* when compared to MGUS patients. It has been reported that high RhoU levels lead to enhanced cell motility, while low RhoU levels are essential for adhesion^47^. It is predictable that RhoU is expressed at high levels in the initial steps of the disease when PCs could be more motile since they are competing for BM niches^52^. With disease progression inside the BM, cells adhere and rely on the microenvironment for their survival, which seems to translate in a cell-contact dependent decrease in *RHOU* expression, hypothesis supported by the in vitro evidence of a *RHOU* down-modulation in MM cell lines cultured in contact with HS-5 stromal cells.

Remarkably, MM patients in high risk TC groups have significantly higher levels of *RHOU* mRNA that positively correlated with the presence of unfavorable cytogenetic alterations; however the sole presence of 1q gain, where RHOU gene is located, was not enough to cause a significant change in its expression. Also, higher levels of *RHOU* expression correlated with increased *CCND2* and decreased cell cycle control and DNA damage repair genes; while RhoU silencing resulted in a decrease in cyclin D2 protein expression and impaired cell cycle progression. Put together, these results suggest that even though most MM patients down-modulate *RHOU* with disease progression, a high expression of this GTPase in late stages of this malignancy could actually be associated with a worse prognosis. We also found that IL-6 stimulus, important for MM survival, resulted in a STAT3-dependent increase in *RHOU* expression in cell lines. Likewise, CD45^+^ U266 cells, that have increased JAK/STAT activation^45,46,^, exhibited a 2.3 times higher MFI for intracytoplasmic RhoU staining. To note, in MM patients *RHOU* expression correlated with the expression of *STAT3* itself, of *MIR21* and of *SOCS3*, confirming that it might be highly dependent on the activation of the STAT3 cascade, as observed in MM cell lines.

RhoU silencing led to a loss of migration capability probably due to the decrease in active JNK, as previous studies suggested: a constitutively active RhoU mutant was able to increase JNK activation in mouse epithelial cells^53^ and RhoU depletion in HelaS3 cells inhibited JNK activating phosphorylation consequently impairing filopodium formation, a type of cell protrusion essential for cell migration^54^. Lastly, we described the effects of IMIDs on the IL-6/STAT3/RhoU/JNK branch. This new generation drugs for MM treatment have been shown to reorganize cells’ cytoskeleton by modulating Rho GTPases in human monocytes^49^. Indeed, we found that lenalidomide commanded an increased activation of STAT3, augmented RhoU expression and amplified JNK phosphorylation. Parallel to the changes in cytoskeleton conformation after lenalidomide, we also observed a decrease in cell cycle progression, probably through a p21 dependent mechanism, in line with what is already known for this drug^51,55,56,^. Consistently and opposite to what was observed with RhoU silencing, lenalidomide led to a high increase in cell motility. The combination of RhoU silencing with lenalidomide treatment led to a loss of cytoskeleton organization that translated in a defective migratory capability, and a further increase in cell cycle arrest. These results give insights into a novel mechanism of action of lenalidomide and prove that this drug can somehow stimulate the activation of STAT3 transcription factor enhancing the expression of its target genes including RHOU, leading to cytoskeletal changes in MM PCs. The increased cell motility upon lenalidomide is consistent with the disruption of microenvironment niche previously described^57,58,^ where less adherent cells are more easily targeted by immune cells or combined chemotherapeutics. Nevertheless, whether or not high RhoU levels indicate an increased propensity towards dissemination of malignant PCs, whether RhoU levels during lenalidomide therapy in MM patients are increased, and whether RhoU high-expressing MM could display different responsiveness to lenalidomide remains to be clarified.

## Electronic supplementary material

Supplementary Material and Methods

Supplementary Table S1

Supplementary Table S2

Supplementary Figure S1

Supplementary Figure S2
