# Supplementary material for: The small GTPase RhoU lays downstream of JAK/STAT signaling and mediates cell migration in multiple myeloma
Source: Blood Cancer J. 2018 Feb 13;8(2):20. doi: 10.1038/s41408-018-0053-z (PMC5811530; doi:10.1038/s41408-018-0053-z)
Supplement: Supplementary file 1 — Supplementary Material and Methods [file 41408_2018_53_MOESM1_ESM.docx]

**SUPPLEMENTARY MATERIAL AND METHODS**

**Western Blot**

Primary antibodies: anti-GAPDH (Millipore, Germany); anti-RhoU (Abcam, UK); anti-phospho-JNK(Thr183/Tyr185) (Cell Signaling, USA); anti-phospho-STAT3(Tyr705) (Cell Signaling, USA); anti-STAT3 (Cell Signaling, USA); anti-cyclinD2 (LSBio, USA); anti-p21 (Santa Cruz, Germany); anti-PARP (Cell Signaling, USA). Secondary antibodies: anti-rabbit IgG HRP-linked antibody (Cell Signaling, USA); HRP labeled goat anti-mouse IgG (KPL, USA).

**qRT-PCR**

RNA isolation and qRT-PCR were performed as previously described ^[59]^ using the following primers: GAPDH (F: AATGGAAATCCCATCACCATCT) (R: CGCCCCACTTGATTTTGG) and *RHOU* (F: GACTCCAACTCTGTGACACTGC) (R: ATGAGGGGCTCACGACACT).

**Cell Cycle**

Cell cycle analysis was performed after Lenalidomide and combination with siRNA, as previously described ^[59]^.

**Kaplan-Meier survival curves**

The prognostic significance of the RHOU mRNA expression in MM was evaluated using publicly available datasets including gene expression data and clinical data at NCBI Gene Expression Omnibus repository #GSE2658 ^[20, 21, 22]^ #GSE57317 ^[23]^. Kaplan-Meier survival curves were analyzed using Graphpad Prism software where p-value and hazard ratio (HR) with 95% confidence intervals were calculated.

**Intracytoplasmic staining**

For intracytoplasmic staining, U266 cells were fixed with cytofix (BD, Italy), permeabilized with Methanol 90% and stained with anti-CD45 APC (BD, Italy), anti-RhoU (Santa Cruz, Germany) and anti-Goat Alexa 488 (Invitrogen, Italy).

**Statistical analysis**

Data were evaluated for their statistical significance with appropriate tests: Student’s t test was used to assess if a mean value of a certain distribution was significantly different from a reference value and when the variances of two groups were significantly different, Welch correction was applied; differences between groups were tested by applying the Analysis of Variance (ANOVA or Kruskal-Wallis test) or Fisher’s exact test; Student’s t test for trend was applied when a trend needed to be verified. P values below 0.05 were considered statistically significant.

Microarray data were globally analyzed by Gene Set Enrichment Analysis (GSEA) and gene sets were considered significant at nominal p-value <0.05 and FDR<25% (0.25). Conventional statistical procedures were applied using standard packages of the R software.

To evaluate the correlation between the expressions of two genes “Pearson product-moment correlation coefficient” was used (p-value≤0.05 and R>0.3).

All analyses were performed using GraphPad Prism 6, Microsoft Excel or R software.

GEP of the 268 samples (accession #GSE66293) was generated using GeneChip® Gene 1.0 ST Array (Affymetrix Inc., Santa Clara, CA) as previously described ^[13]^. Supervised analyses were performed using Significant Analysis of Microarrays software (SAM version 4.00; Excel front-end publicly available at <http://www-stat.stanford.edu/tibs/SAM/index.html>) ^[60]^. Differentially expressed gene list was chosen at a statistical significance of q-value=0 (median FDR=0, 90th perc FDR=0) and it was submitted to the DAVID Bioinformatics Resources 6.8 (https://david.ncifcrf.gov/) for functional annotation.
