# Supplementary material for: The small GTPase RhoU lays downstream of JAK/STAT signaling and mediates cell migration in multiple myeloma
Source: Blood Cancer J. 2018 Feb 13;8(2):20. doi: 10.1038/s41408-018-0053-z (PMC5811530; doi:10.1038/s41408-018-0053-z)
Supplement: Supplementary file 2 — Supplementary Table S1 [file 41408_2018_53_MOESM2_ESM.docx]

**Supplementary Table S1:** List of 557 differentially expressed genes in RHOU quartile I vs quartile IV by SAM analysis at high stringency level (median FDR=0, 90th perc FDR=0). Genes are ordered according to SAM score, the fold change (FC) is also reported.

| **Gene Name** | **Score(d)** | **FC** |  | **Gene Name** | **Score(d)** | **FC** |  | **Gene Name** | **Score(d)** | **FC** |
| --- | --- | --- | --- | --- | --- | --- | --- | --- | --- | --- |
| RHOU | 10.17 | 3.94 |  | UBE2O | -4.33 | 0.7 |  | ORAI2 | -3.66 | 0.57 |
| SOCS3 | 7.54 | 4.44 |  | ZBTB40 | -4.32 | 0.55 |  | SHPK | -3.66 | 0.77 |
| C1orf21 | 7.07 | 4.38 |  | SYNJ2BP | -4.32 | 0.56 |  | CCDC53 | -3.66 | 0.65 |
| PELI2 | 5.91 | 2.23 |  | GPRIN3 | -4.32 | 0.36 |  | DFFB | -3.66 | 0.79 |
| BOC | 5.68 | 1.7 |  | MLH1 | -4.31 | 0.65 |  | ZNF25 | -3.66 | 0.68 |
| BACH2 | 5.57 | 2.45 |  | SRBD1 | -4.31 | 0.56 |  | NBN | -3.65 | 0.66 |
| GPRC5D | 5.48 | 3.51 |  | KMO | -4.3 | 0.36 |  | IPO13 | -3.65 | 0.71 |
| SESTD1 | 5.35 | 3.03 |  | METTL4 | -4.3 | 0.56 |  | ATP6V0A1 | -3.65 | 0.61 |
| ARL4C | 5.34 | 3.02 |  | FBXO8 | -4.3 | 0.7 |  | REV1 | -3.65 | 0.73 |
| GNAL | 5.33 | 2.69 |  | PARN | -4.3 | 0.69 |  | PLCG1 | -3.64 | 0.7 |
| NCAM1 | 5.26 | 5.85 |  | CARF | -4.28 | 0.62 |  | AMPD1 | -3.64 | 0.49 |
| OCLN | 5.22 | 3.6 |  | THAP6 | -4.26 | 0.64 |  | VPS35 | -3.64 | 0.7 |
| SVIL | 5.19 | 2.23 |  | PTPLAD2 | -4.26 | 0.44 |  | POLA2 | -3.64 | 0.66 |
| SGK1 | 5.18 | 2.21 |  | ZNF589 | -4.26 | 0.64 |  | POLE | -3.63 | 0.71 |
| WARS | 5.18 | 2.11 |  | RAD51B | -4.26 | 0.6 |  | FICD | -3.63 | 0.66 |
| CYTH3 | 5.12 | 1.36 |  | PACRGL | -4.25 | 0.67 |  | PTER | -3.63 | 0.74 |
| KDM5B | 5.06 | 2.1 |  | PRIMPOL | -4.24 | 0.61 |  | MINPP1 | -3.63 | 0.58 |
| PNP | 5.01 | 2.65 |  | CDK10 | -4.24 | 0.71 |  | CCDC110 | -3.62 | 0.6 |
| SCN9A | 4.91 | 2.63 |  | PCED1B | -4.24 | 0.54 |  | IFI6 | -3.61 | 0.39 |
| ZNF460 | 4.9 | 1.56 |  | CTC1 | -4.24 | 0.66 |  | SP140L | -3.61 | 0.71 |
| SLCO3A1 | 4.87 | 3.45 |  | ZNF33A | -4.24 | 0.61 |  | ERLIN1 | -3.61 | 0.59 |
| NOS1AP | 4.81 | 1.38 |  | CCR1 | -4.23 | 0.37 |  | THOC5 | -3.61 | 0.7 |
| PELI1 | 4.75 | 1.95 |  | CEP104 | -4.22 | 0.69 |  | SNRNP40 | -3.61 | 0.75 |
| TPBG | 4.72 | 1.5 |  | MTRF1 | -4.22 | 0.64 |  | KIZ | -3.61 | 0.72 |
| C10orf11 | 4.68 | 1.67 |  | RERE | -4.21 | 0.59 |  | ANAPC7 | -3.61 | 0.65 |
| GRIN2B | 4.68 | 1.9 |  | TMCC1 | -4.21 | 0.7 |  | FOXRED2 | -3.6 | 0.62 |
| PVR | 4.68 | 1.37 |  | MAML2 | -4.21 | 0.34 |  | LDLRAP1 | -3.6 | 0.67 |
| MIR21 | 4.66 | 3.23 |  | PLAC8 | -4.21 | 0.42 |  | CDKL5 | -3.6 | 0.61 |
| C11orf63 | 4.65 | 2.07 |  | C16orf58 | -4.2 | 0.74 |  | AMZ2P1 | -3.6 | 0.64 |
| CAPN2 | 4.64 | 1.77 |  | PSMA3 | -4.2 | 0.66 |  | C14orf159 | -3.6 | 0.66 |
| SBNO2 | 4.63 | 1.72 |  | LOC100133315 | -4.2 | 0.49 |  | EBAG9 | -3.59 | 0.65 |
| ZBTB43 | 4.63 | 1.97 |  | FMNL2 | -4.19 | 0.51 |  | ELP3 | -3.59 | 0.64 |
| MUC1 | 4.63 | 2.41 |  | PPIL2 | -4.19 | 0.64 |  | UBE3B | -3.59 | 0.74 |
| PLEKHO2 | 4.59 | 1.39 |  | LIMD1 | -4.19 | 0.56 |  | LIN54 | -3.59 | 0.74 |
| TMEM173 | 4.56 | 2.3 |  | PGD | -4.18 | 0.7 |  | APOBEC3D | -3.59 | 0.65 |
| MARK1 | 4.55 | 1.85 |  | TBCK | -4.18 | 0.55 |  | UFL1 | -3.59 | 0.7 |
| C10orf10 | 4.55 | 3.15 |  | ZNRD1-AS1 | -4.18 | 0.54 |  | MSL3 | -3.59 | 0.71 |
| SLAMF1 | 4.52 | 4.03 |  | TRAPPC11 | -4.18 | 0.66 |  | DET1 | -3.59 | 0.63 |
| AHR | 4.52 | 3.09 |  | NUP62CL | -4.18 | 0.6 |  | CSGALNACT1 | -3.59 | 0.45 |
| HK1 | 4.49 | 1.54 |  | ago.03 | -4.18 | 0.66 |  | SASS6 | -3.58 | 0.58 |
| S100A10 | 4.49 | 3.03 |  | HIST1H4L | -4.17 | 0.56 |  | ZNF780A | -3.58 | 0.7 |
| TLDC2 | 4.49 | 1.62 |  | RHBDF2 | -4.15 | 0.72 |  | RAB28 | -3.58 | 0.69 |
| CD48 | 4.48 | 2.02 |  | DDX17 | -4.15 | 0.58 |  | DUSP6 | -3.58 | 0.39 |
| WSCD1 | 4.45 | 1.89 |  | NFYC | -4.14 | 0.72 |  | ZMYM4 | -3.58 | 0.68 |
| BACE2 | 4.43 | 1.84 |  | ZNF860 | -4.14 | 0.44 |  | HERC6 | -3.57 | 0.45 |
| PPM1H | 4.4 | 1.38 |  | MAGOHB | -4.14 | 0.69 |  | ANKEF1 | -3.57 | 0.63 |
| MAL | 4.4 | 3.64 |  | IFIT1 | -4.14 | 0.29 |  | C4orf29 | -3.57 | 0.68 |
| LDHA | 4.37 | 1.6 |  | TRNT1 | -4.14 | 0.63 |  | FAM118B | -3.57 | 0.64 |
| GEM | 4.36 | 2.5 |  | TCEANC | -4.13 | 0.6 |  | PIP5K1B | -3.57 | 0.54 |
| DOCK11 | 4.33 | 1.97 |  | E2F7 | -4.12 | 0.5 |  | KLHDC1 | -3.57 | 0.58 |
| SH3RF1 | 4.32 | 2.12 |  | GVINP1 | -4.12 | 0.57 |  | TFEC | -3.56 | 0.37 |
| PARP15 | 4.3 | 3.72 |  | POLR2K | -4.12 | 0.63 |  | TK2 | -3.56 | 0.69 |
| SOX4 | 4.3 | 1.6 |  | LIAS | -4.11 | 0.68 |  | KRIT1 | -3.56 | 0.73 |
| CDYL2 | 4.26 | 1.51 |  | TNFSF8 | -4.11 | 0.29 |  | CCDC82 | -3.56 | 0.69 |
| ASB7 | 4.25 | 1.53 |  | DEPDC5 | -4.11 | 0.65 |  | ZNF721 | -3.56 | 0.53 |
| STAT3 | 4.25 | 1.82 |  | SMARCAD1 | -4.1 | 0.59 |  | KIAA1919 | -3.56 | 0.71 |
| EPS8 | 4.25 | 2.51 |  | HIST1H3I | -4.1 | 0.45 |  | ZNF143 | -3.55 | 0.7 |
| RIOK3 | 4.22 | 1.56 |  | VIPAS39 | -4.1 | 0.64 |  | OSBPL10 | -3.55 | 0.52 |
| HSD3B7 | 4.21 | 1.34 |  | EEA1 | -4.08 | 0.63 |  | ROCK2 | -3.55 | 0.65 |
| SSBP4 | 4.21 | 1.33 |  | PRMT9 | -4.08 | 0.69 |  | ODF2L | -3.55 | 0.61 |
| MTUS2-AS1 | 4.21 | 1.45 |  | WFS1 | -4.08 | 0.58 |  | ANKRD26 | -3.55 | 0.65 |
| CCND2 | 4.19 | 6.67 |  | OCIAD1 | -4.07 | 0.73 |  | JMJD8 | -3.55 | 0.61 |
| GALNT3 | 4.19 | 2.61 |  | FRYL | -4.06 | 0.67 |  | NUDT7 | -3.54 | 0.59 |
| CDC42SE1 | 4.18 | 1.56 |  | CBY1 | -4.04 | 0.74 |  | LOC102724873 | -3.54 | 0.67 |
| TSPAN7 | 4.16 | 4.98 |  | DCP1B | -4.03 | 0.62 |  | GPAM | -3.54 | 0.71 |
| GADD45B | 4.16 | 2.02 |  | PPIL3 | -4.03 | 0.65 |  | C2CD5 | -3.54 | 0.68 |
| ZNF281 | 4.15 | 1.45 |  | TUBD1 | -4.03 | 0.59 |  | BLNK | -3.53 | 0.58 |
| MAP1B | 4.15 | 2 |  | BET1 | -4.03 | 0.63 |  | RAD50 | -3.53 | 0.67 |
| ELOVL4 | 4.12 | 2.55 |  | ZNF215 | -4.03 | 0.53 |  | ZNF37A | -3.53 | 0.75 |
| LRIG1 | 4.12 | 2.17 |  | CEP290 | -4.03 | 0.68 |  | EARS2 | -3.53 | 0.77 |
| YBX3 | 4.1 | 3.1 |  | CNNM2 | -4.03 | 0.72 |  | ACAD10 | -3.53 | 0.68 |
| PLAGL1 | 4.1 | 1.8 |  | TBC1D5 | -4.03 | 0.63 |  | RBFOX2 | -3.52 | 0.63 |
| CAPN5 | 4.1 | 1.77 |  | PEF1 | -4.02 | 0.74 |  | LINC00471 | -3.52 | 0.71 |
| TNFRSF11A | 4.09 | 1.49 |  | APOBEC3C | -4.01 | 0.62 |  | PDDC1 | -3.52 | 0.78 |
| ATP13A3 | 4.08 | 1.52 |  | GPR174 | -4.01 | 0.52 |  | FCHSD2 | -3.52 | 0.59 |
| SLC39A10 | 4.05 | 1.65 |  | LRIF1 | -4 | 0.59 |  | IPP | -3.52 | 0.64 |
| NARF | 4.04 | 1.47 |  | KIAA0586 | -3.99 | 0.63 |  | TTC27 | -3.52 | 0.66 |
| ZNF697 | 4.04 | 1.27 |  | ZDHHC14 | -3.98 | 0.52 |  | PMS1 | -3.51 | 0.67 |
| ZNF503 | 4.04 | 1.25 |  | NUP43 | -3.98 | 0.54 |  | OAS2 | -3.51 | 0.56 |
| SLC7A1 | 4.04 | 1.6 |  | SEC31A | -3.97 | 0.66 |  | SNAPC1 | -3.51 | 0.68 |
| MAGI1 | 4.03 | 1.63 |  | PRKACB | -3.95 | 0.54 |  | DUS2 | -3.51 | 0.64 |
| KLF13 | 4.02 | 1.48 |  | FAM192A | -3.95 | 0.73 |  | MRPS31 | -3.51 | 0.72 |
| RNF130 | 4.02 | 2.69 |  | ZFP90 | -3.95 | 0.73 |  | CEP97 | -3.51 | 0.59 |
| BMP8B | 4.01 | 1.58 |  | PAPPA-AS1 | -3.94 | 0.64 |  | PEX3 | -3.51 | 0.67 |
| BCL2L10 | 4.01 | 1.29 |  | TOE1 | -3.94 | 0.71 |  | COX15 | -3.51 | 0.66 |
| RAP2B | 4.01 | 1.54 |  | STAM2 | -3.94 | 0.67 |  | RBL1 | -3.51 | 0.58 |
| MYO1E | 4 | 2.25 |  | TTI1 | -3.94 | 0.66 |  | TMEM135 | -3.51 | 0.66 |
| ROBO3 | 3.99 | 1.25 |  | CARD8 | -3.94 | 0.67 |  | MACC1 | -3.51 | 0.55 |
| UCHL1 | 3.99 | 4.2 |  | ATG10 | -3.94 | 0.7 |  | GOLPH3L | -3.5 | 0.51 |
| NKD2 | 3.99 | 1.19 |  | HP1BP3 | -3.93 | 0.74 |  | HEMK1 | -3.5 | 0.74 |
| PTPN1 | 3.96 | 1.61 |  | TRMT1L | -3.93 | 0.56 |  | DNAJA3 | -3.5 | 0.69 |
| ANKRD18A | 3.93 | 1.48 |  | DAPP1 | -3.93 | 0.56 |  | FIG4 | -3.5 | 0.63 |
| S1PR4 | 3.91 | 1.79 |  | IFI44 | -3.93 | 0.31 |  | GOLGA5 | -3.5 | 0.7 |
| TAP1 | 3.91 | 1.7 |  | IQCG | -3.93 | 0.64 |  | NOC3L | -3.5 | 0.59 |
| SHB | 3.88 | 1.32 |  | DENND2D | -3.92 | 0.68 |  | NSUN4 | -3.5 | 0.78 |
| TPPP | 3.86 | 1.29 |  | NF1 | -3.92 | 0.65 |  | CBR4 | -3.49 | 0.64 |
| JPH1 | 3.81 | 1.22 |  | TRIM55 | -3.92 | 0.49 |  | SCNN1B | -3.49 | 0.48 |
| SH3GL1 | 3.8 | 1.43 |  | MOSPD2 | -3.92 | 0.55 |  | NUDT6 | -3.49 | 0.77 |
| FAM78B | 3.8 | 1.36 |  | CHM | -3.91 | 0.68 |  | PANK4 | -3.49 | 0.8 |
| SYT3 | 3.8 | 1.27 |  | IFT46 | -3.91 | 0.59 |  | NOL12 | -3.48 | 0.8 |
| FBXO16 | -6.31 | 0.36 |  | ACTR8 | -3.91 | 0.68 |  | FAM185A | -3.48 | 0.6 |
| ABCG2 | -5.67 | 0.21 |  | MOB3A | -3.91 | 0.49 |  | BMP2K | -3.48 | 0.5 |
| C20orf196 | -5.62 | 0.68 |  | ACADM | -3.91 | 0.53 |  | ADAT1 | -3.48 | 0.72 |
| ATP10D | -5.51 | 0.44 |  | PRDM5 | -3.91 | 0.39 |  | IRF2 | -3.48 | 0.58 |
| C11orf54 | -5.42 | 0.55 |  | SWT1 | -3.91 | 0.68 |  | REEP3 | -3.48 | 0.77 |
| ZNF248 | -5.42 | 0.52 |  | TRAC | -3.9 | 0.43 |  | SF3B3 | -3.48 | 0.69 |
| MAN1A1 | -5.41 | 0.55 |  | CASP8AP2 | -3.9 | 0.65 |  | HMG20A | -3.47 | 0.71 |
| SFMBT2 | -5.36 | 0.53 |  | MLYCD | -3.89 | 0.7 |  | ATP6V1E1 | -3.47 | 0.69 |
| SLC44A2 | -5.17 | 0.37 |  | PARM1 | -3.89 | 0.45 |  | TMEM194B | -3.47 | 0.71 |
| NHLRC2 | -5.17 | 0.54 |  | STXBP4 | -3.89 | 0.69 |  | CNOT1 | -3.47 | 0.71 |
| CHMP5 | -5.16 | 0.5 |  | FTO | -3.88 | 0.7 |  | OAS1 | -3.47 | 0.53 |
| PDK1 | -5.14 | 0.56 |  | ARL1 | -3.88 | 0.7 |  | UGDH | -3.46 | 0.64 |
| HELQ | -5.12 | 0.62 |  | HSH2D | -3.88 | 0.56 |  | XBP1 | -3.46 | 0.7 |
| NARS2 | -5.04 | 0.56 |  | USO1 | -3.87 | 0.68 |  | BTN3A3 | -3.46 | 0.62 |
| BICD1 | -4.98 | 0.57 |  | DHDDS | -3.87 | 0.7 |  | BBS2 | -3.45 | 0.67 |
| AMN1 | -4.95 | 0.57 |  | SMYD4 | -3.86 | 0.76 |  | SZT2 | -3.45 | 0.76 |
| TXLNB | -4.94 | 0.45 |  | TRIM66 | -3.85 | 0.69 |  | MAP3K13 | -3.45 | 0.62 |
| FAM111A | -4.92 | 0.54 |  | COQ10A | -3.85 | 0.65 |  | ATP7A | -3.45 | 0.71 |
| NAGK | -4.91 | 0.57 |  | POLR3K | -3.85 | 0.64 |  | SS18L1 | -3.45 | 0.84 |
| STAP1 | -4.9 | 0.3 |  | SLC25A17 | -3.84 | 0.73 |  | AHSA2 | -3.44 | 0.67 |
| LARP7 | -4.88 | 0.68 |  | MTFR1 | -3.83 | 0.64 |  | ZCCHC17 | -3.44 | 0.76 |
| SLX4IP | -4.87 | 0.5 |  | RCBTB2 | -3.82 | 0.48 |  | ZNF646 | -3.44 | 0.78 |
| RBBP4 | -4.84 | 0.64 |  | NIF3L1 | -3.82 | 0.59 |  | TAF6L | -3.44 | 0.79 |
| PLCG2 | -4.84 | 0.46 |  | UNC50 | -3.82 | 0.74 |  | ALDH5A1 | -3.44 | 0.62 |
| WDR64 | -4.83 | 0.45 |  | USP54 | -3.82 | 0.69 |  | TRIP11 | -3.44 | 0.58 |
| PLCL2 | -4.82 | 0.54 |  | SSBP2 | -3.82 | 0.48 |  | HPS4 | -3.43 | 0.73 |
| FGD4 | -4.8 | 0.35 |  | PPME1 | -3.81 | 0.7 |  | COQ2 | -3.43 | 0.72 |
| FAM69A | -4.8 | 0.48 |  | STAU2 | -3.81 | 0.7 |  | TRIT1 | -3.43 | 0.72 |
| PTPRJ | -4.79 | 0.4 |  | RBM4B | -3.8 | 0.66 |  | INVS | -3.43 | 0.66 |
| SPATA5 | -4.79 | 0.58 |  | EMC2 | -3.8 | 0.61 |  | BCL2L13 | -3.43 | 0.72 |
| PDP2 | -4.79 | 0.59 |  | MCM8 | -3.8 | 0.54 |  | FMNL3 | -3.43 | 0.66 |
| KIAA1551 | -4.77 | 0.58 |  | BTN3A1 | -3.79 | 0.63 |  | PIGV | -3.43 | 0.71 |
| ITGB3BP | -4.75 | 0.59 |  | INTS2 | -3.79 | 0.6 |  | SYK | -3.43 | 0.54 |
| EDRF1 | -4.74 | 0.65 |  | CRYZ | -3.79 | 0.55 |  | C17orf75 | -3.42 | 0.66 |
| CHST15 | -4.73 | 0.63 |  | TP73-AS1 | -3.79 | 0.63 |  | ARRDC1-AS1 | -3.42 | 0.65 |
| ATM | -4.7 | 0.56 |  | EPB41 | -3.79 | 0.75 |  | GIT2 | -3.42 | 0.65 |
| ZMYM1 | -4.7 | 0.59 |  | SENP8 | -3.79 | 0.74 |  | SYNRG | -3.42 | 0.63 |
| C10orf76 | -4.67 | 0.66 |  | TANGO6 | -3.79 | 0.7 |  | DOCK7 | -3.42 | 0.71 |
| PPM1K | -4.67 | 0.53 |  | ORC3 | -3.78 | 0.61 |  | BLZF1 | -3.42 | 0.63 |
| TMEM80 | -4.66 | 0.63 |  | VPS45 | -3.78 | 0.58 |  | AMT | -3.42 | 0.71 |
| THAP9 | -4.66 | 0.63 |  | BTRC | -3.78 | 0.72 |  | NEK1 | -3.42 | 0.67 |
| C10orf88 | -4.65 | 0.59 |  | WDR89 | -3.78 | 0.55 |  | NAA40 | -3.41 | 0.72 |
| DDX60L | -4.64 | 0.37 |  | NSUN6 | -3.77 | 0.61 |  | S100PBP | -3.41 | 0.6 |
| KDM8 | -4.64 | 0.73 |  | COL24A1 | -3.77 | 0.64 |  | C8orf44 | -3.41 | 0.69 |
| PRDX1 | -4.63 | 0.58 |  | TLR6 | -3.77 | 0.52 |  | SASH3 | -3.41 | 0.65 |
| CCR2 | -4.62 | 0.26 |  | PLXNB2 | -3.77 | 0.5 |  | ZMYND8 | -3.41 | 0.66 |
| GIN1 | -4.62 | 0.61 |  | COG4 | -3.77 | 0.63 |  | SLC25A11 | -3.41 | 0.74 |
| ANAPC4 | -4.62 | 0.67 |  | CASP9 | -3.77 | 0.73 |  | NEK8 | -3.41 | 0.78 |
| NUP160 | -4.62 | 0.55 |  | OGG1 | -3.77 | 0.67 |  | WBP2 | -3.4 | 0.76 |
| CBX5 | -4.6 | 0.49 |  | ATP8A1 | -3.77 | 0.6 |  | AAAS | -3.4 | 0.71 |
| EFCAB7 | -4.6 | 0.64 |  | CTBP1-AS2 | -3.76 | 0.62 |  | LRRC37A4P | -3.4 | 0.63 |
| STX17 | -4.59 | 0.62 |  | MILR1 | -3.76 | 0.5 |  | NDUFAF7 | -3.4 | 0.69 |
| CBFA2T2 | -4.58 | 0.67 |  | GPCPD1 | -3.76 | 0.63 |  | NME6 | -3.4 | 0.76 |
| DNAJC16 | -4.58 | 0.7 |  | ADAL | -3.76 | 0.58 |  | LIPT1 | -3.39 | 0.7 |
| BBS7 | -4.58 | 0.66 |  | MED31 | -3.76 | 0.7 |  | ACACB | -3.39 | 0.56 |
| UFSP2 | -4.58 | 0.59 |  | CYB5B | -3.76 | 0.68 |  | KIF18A | -3.39 | 0.52 |
| CTNS | -4.58 | 0.57 |  | UTP20 | -3.76 | 0.66 |  | SMC2 | -3.38 | 0.56 |
| INTS4 | -4.57 | 0.58 |  | RNF214 | -3.75 | 0.65 |  | RPP38 | -3.38 | 0.64 |
| DHTKD1 | -4.57 | 0.61 |  | MIR186 | -3.75 | 0.59 |  | GALK2 | -3.38 | 0.66 |
| PYGB | -4.57 | 0.63 |  | MMACHC | -3.75 | 0.65 |  | ORC4 | -3.37 | 0.72 |
| MGA | -4.55 | 0.57 |  | SPG11 | -3.74 | 0.65 |  | AKTIP | -3.37 | 0.7 |
| CD28 | -4.55 | 0.26 |  | SEC14L1 | -3.74 | 0.71 |  | DUSP22 | -3.37 | 0.64 |
| DDX60 | -4.55 | 0.38 |  | COG5 | -3.74 | 0.73 |  | MTOR | -3.37 | 0.75 |
| TPCN1 | -4.53 | 0.61 |  | GNPTAB | -3.74 | 0.68 |  | DDB2 | -3.37 | 0.71 |
| KIAA1407 | -4.53 | 0.51 |  | TLR10 | -3.74 | 0.38 |  | PRPF8 | -3.37 | 0.74 |
| ING4 | -4.52 | 0.67 |  | DENND6B | -3.74 | 0.65 |  | HIST1H4F | -3.37 | 0.58 |
| KIAA0319L | -4.52 | 0.69 |  | LAMA2 | -3.74 | 0.42 |  | ZNF75A | -3.37 | 0.67 |
| MFSD8 | -4.51 | 0.66 |  | AACS | -3.73 | 0.73 |  | SGMS1 | -3.36 | 0.78 |
| METTL14 | -4.51 | 0.64 |  | TSSC1 | -3.73 | 0.71 |  | WDHD1 | -3.36 | 0.62 |
| TMCO4 | -4.51 | 0.71 |  | SPG7 | -3.72 | 0.76 |  | MKL2 | -3.35 | 0.7 |
| HEATR5A | -4.49 | 0.59 |  | TBCD | -3.72 | 0.79 |  | EYA3 | -3.35 | 0.65 |
| ATG4C | -4.49 | 0.49 |  | TTC9C | -3.71 | 0.68 |  | ZNF518A | -3.35 | 0.65 |
| GOSR2 | -4.48 | 0.72 |  | XPNPEP3 | -3.71 | 0.73 |  | EXOSC10 | -3.35 | 0.79 |
| DOCK8 | -4.45 | 0.57 |  | EHBP1 | -3.7 | 0.64 |  | ACIN1 | -3.35 | 0.76 |
| APITD1 | -4.44 | 0.69 |  | EXOSC8 | -3.7 | 0.65 |  | P2RX4 | -3.35 | 0.73 |
| SEC23B | -4.41 | 0.61 |  | BRCA1 | -3.69 | 0.56 |  | MPV17L2 | -3.35 | 0.65 |
| YAE1D1 | -4.4 | 0.69 |  | DCLRE1A | -3.69 | 0.59 |  | OSGEPL1 | -3.35 | 0.64 |
| TADA2A | -4.39 | 0.63 |  | STARD13 | -3.68 | 0.6 |  | LRRCC1 | -3.34 | 0.58 |
| APOBEC3G | -4.39 | 0.49 |  | METTL3 | -3.68 | 0.66 |  | PRR14L | -3.34 | 0.72 |
| TRANK1 | -4.37 | 0.47 |  | DCLRE1C | -3.67 | 0.68 |  | N4BP1 | -3.34 | 0.73 |
| FAM13A | -4.36 | 0.48 |  | MYEOV | -3.67 | 0.43 |  | LIG3 | -3.33 | 0.76 |
| MED22 | -4.35 | 0.74 |  | AP3M1 | -3.67 | 0.69 |  | NFXL1 | -3.33 | 0.73 |
| PALB2 | -4.35 | 0.66 |  | ANKFY1 | -3.67 | 0.72 |  | C17orf80 | -3.33 | 0.69 |
| CKLF | -4.35 | 0.64 |  | GPR114 | -3.67 | 0.55 |  | USP6NL | -3.33 | 0.54 |
| PARP2 | -4.34 | 0.66 |  | TFB1M | -3.67 | 0.59 |  | CHTF8 | -3.33 | 0.79 |
| FRRS1 | -4.33 | 0.58 |  | GMPPA | -3.67 | 0.7 |  |  |  |  |
