# Supplementary material for: The small GTPase RhoU lays downstream of JAK/STAT signaling and mediates cell migration in multiple myeloma
Source: Blood Cancer J. 2018 Feb 13;8(2):20. doi: 10.1038/s41408-018-0053-z (PMC5811530; doi:10.1038/s41408-018-0053-z)
Supplement: Supplementary file 3 — Supplementary Table S2 [file 41408_2018_53_MOESM3_ESM.docx]

**Supplementary Table S2:** List of the significantly enriched functional annotation clusters for the 557 differentially expressed genes, by DAVID Bioinformatics Resources 6.8. Twelve clusters with Enrichment Score greater than 1.3 (p-value <0.05) and 6 significant pathways (p-value <0.05).

| **Category** | **Term** | **Count** | **PValue** | **Genes** |
| --- | --- | --- | --- | --- |
| Annotation Cluster 1 | Enrichment Score: 4.5095359761632245 |  | | |
| GOTERM_CC_FAT | GO:0070013~intracellular organelle lumen | 74 | 7.35E-06 | ITGB3BP, RPP38, NBN, PDP2, E2F7, UCHL1, INTS2, APOBEC3G, MED22, CBX5, RBM4B, BLZF1, PARN, MCM8, INTS4, NARS2, NARF, GIT2, ACIN1, ELP3, MINPP1, EXOSC8, RBBP4, ACADM, TADA2A, DFFB, RBL1, POLE, ANAPC4, LIG3, HMG20A, CBR4, TAF6L, ZCCHC17, EARS2, ATG4C, PPM1K, LARP7, DDB2, ANAPC7, TFB1M, NOL12, SMARCAD1, ING4, HIST1H4L, POLR2K, PPIL2, NOC3L, NFYC, POLA2, STAU2, EXOSC10, TOE1, METTL3, STX17, PRPF8, HIST1H4F, LIAS, WDHD1, DNAJA3, PDK1, POLR3K, MAML2, FOXRED2, CBY1, STAT3, ATM, ZBTB43, BRCA1, MED31, OGG1, UTP20, PARP2, RERE, DUSP6 |
| Annotation Cluster 2 | Enrichment Score: 2.669358453606318 |  | | |
| GOTERM_MF_FAT | GO:0001882~nucleoside binding | 66 | 0.001 | SPG7, HELQ, PIP5K1B, MLH1, ATP10D, PNP, WARS, DDX17, MCM8, NARS2, DDX60, CDK10, CHST15, PRKACB, GOLGA5, PMS1, SYK, SGK1, ACADM, MAGI1, ROCK2, LIG3, GEM, RAD50, CDKL5, MARK1, TBCK, EARS2, NME6, PANK4, UBE2O, RIOK3, BMP2K, NEK8, MAP3K13, SMARCAD1, NEK1, HK1, OAS1, KMO, SHPK, OAS2, NAGK, TK2, C10ORF88, GALK2, TAP1, DDX60L, SPATA5, PDK1, MYO1E, FOXRED2, KIF18A, ACACB, ATP13A3, SMC2, ATM, ABCG2, TRNT1, ATP7A, P2RX4, MTOR, AACS, ACAD10, TRIT1, ATP8A1 |
| Annotation Cluster 3 | Enrichment Score: 2.2485259089563794 |  | | |
| GOTERM_BP_FAT | GO:0006259~DNA metabolic process | 27 | 0.001 | ING4, NBN, HSD3B7, MLH1, POLA2, TK2, GIN1, MCM8, PMS1, DNAJA3, HEMK1, RBBP4, REV1, CHTF8, DFFB, POLE, LIG3, RAD50, BRCA1, ATM, LOC100133315, DCLRE1C, EYA3, DCLRE1A, DDB2, OGG1, PARP2 |
| Annotation Cluster 4 | Enrichment Score: 2.1688130566912833 |  | | |
| GOTERM_BP_FAT | GO:0046907~intracellular transport | 34 | 7.43E-04 | SPG7, SEC31A, NUP160, CHMP5, STAM2, HPS4, UCHL1, BET1, EEA1, PEX3, POLA2, AP3M1, STX17, CHM, VPS35, GOLGA5, ARL1, SYNRG, STAP1, IPO13, VPS45, MYO1E, STXBP4, TRNT1, COG4, COG5, AAAS, ATG4C, AKTIP, USO1, GOSR2, THOC5, SEC23B, RERE |
| Annotation Cluster 5 | Enrichment Score: 2.076171522968553 |  | | |
| GOTERM_CC_FAT | GO:0044431~Golgi apparatus part | 19 | 4.11E-04 | ARL1, SYNRG, SEC31A, UNC50, PPIL2, BET1, SGMS1, CBY1, MAN1A1, LDLRAP1, ATP7A, CSGALNACT1, COG4, BLZF1, COG5, USO1, GOSR2, GOLGA5, SEC23B |
| Annotation Cluster 6 | Enrichment Score: 1.8690598435271313 |  | | |
| GOTERM_CC_FAT | GO:0044431~Golgi apparatus part | 19 | 4.11E-04 | ARL1, SYNRG, SEC31A, UNC50, PPIL2, BET1, SGMS1, CBY1, MAN1A1, LDLRAP1, ATP7A, CSGALNACT1, COG4, BLZF1, COG5, USO1, GOSR2, GOLGA5, SEC23B |
| Annotation Cluster 7 | Enrichment Score: 1.5390173893922316 |  | | |
| GOTERM_MF_FAT | GO:0030674~protein binding, bridging | 9 | 0.004 | SHB, STAP1, EPS8, GOLGA5, LDLRAP1, RAD50, CBX5, BLNK, HSH2D |
| Annotation Cluster 8 | Enrichment Score: 1.4400504582290863 |  | | |
| GOTERM_CC_FAT | GO:0005694~chromosome | 21 | 0.01 | ITGB3BP, HIST1H4L, NBN, RBBP4, TADA2A, NUP160, HP1BP3, MLH1, POLA2, SMC2, RAD50, BRCA1, CBX5, MSL3, TRIM66, APITD1, CDYL2, HIST1H4F, LIN54, NUP43, SS18L1, HIST1H3I |
| Annotation Cluster 9 | Enrichment Score: 1.4399326702832158 |  | | |
| GOTERM_MF_FAT | GO:0016504~peptidase activator activity | 5 | 0.003 | CARD8, CASP8AP2, MAL, BCL2L13, EBAG9 |
| Annotation Cluster 10 | Enrichment Score: 1.3668886035402041 |  | | |
| GOTERM_CC_FAT | GO:0043228~non-membrane-bounded organelle | 82 | 0.013 | ITGB3BP, RPP38, NBN, HP1BP3, UCHL1, MLH1, APOBEC3G, RHOU, MRPS31, PNP, CBX5, RBM4B, PARN, GRIN2B, NARF, CEP290, ACIN1, ACTR8, SS18L1, ELP3, EXOSC8, RBBP4, TADA2A, ROCK2, HMG20A, ZCCHC17, RAD50, MARK1, BICD1, BBS2, SASS6, APITD1, ATG4C, KRIT1, TBCD, TPPP, PSMA3, LIN54, TFB1M, NUP43, NOL12, CEP97, SMARCAD1, HIST1H4L, NUP160, HSD3B7, THAP6, NOC3L, LRRCC1, CDC42SE1, POLA2, STAU2, EXOSC10, TRIM66, TOE1, CDYL2, STX17, HIST1H4F, LIAS, WDHD1, TRIP11, DNAJA3, FGD4, IPP, EPB41, MYO1E, MAP1B, KIF18A, SMC2, STAT3, ATM, ZBTB43, BRCA1, MSL3, TRIM55, P2RX4, EPS8, INVS, SVIL, TUBD1, UTP20, PARP2, HIST1H3I |
| Annotation Cluster 11 | Enrichment Score: 1.3626300849668067 |  | | |
| GOTERM_BP_FAT | GO:0051276~chromosome organization | 24 | 0.008 | SMARCAD1, ING4, HIST1H4L, NBN, RBBP4, TADA2A, HP1BP3, DFFB, RBL1, KIF18A, HMG20A, TAF6L, SMC2, RAD50, CBX5, DCLRE1C, EYA3, MSL3, CDYL2, PRDM5, HIST1H4F, ACIN1, KDM5B, RERE, HIST1H3I |
| Annotation Cluster 12 | Enrichment Score: 1.3387067733256162 |  | | |
| GOTERM_BP_FAT | GO:0001775~cell activation | 17 | 0.006 | NBN, SBNO2, MLH1, SOX4, TLR6, SLAMF1, HSH2D, CD48, ATP7A, DCLRE1C, P2RX4, SHB, PLCG2, DNAJA3, SYK, BLNK, CD28 |

| **Category** | **Term** | **Count** | **P-Value** | **Genes** |
| --- | --- | --- | --- | --- |
| BIOCARTA | h_atmPathway: ATM Signaling Pathway | 4 | 0.01 | NBN, ATM, RAD50, BRCA1 |
| BIOCARTA | h_atrbrcaPathway: Role of BRCA1, BRCA2 and ATR in Cancer Susceptibility | 4 | 0.02 | NBN, ATM, RAD50, BRCA1 |
| KEGG_PATHWAY | hsa04120:Ubiquitin mediated proteolysis | 10 | 0.01 | UBE2O, UBE3B, SOCS3, BTRC, PPIL2, ANAPC4, DDB2, DET1, ANAPC7, BRCA1 |
| KEGG_PATHWAY | hsa00520:Amino sugar and nucleotide sugar metabolism | 5 | 0.03 | GALK2, GMPPA, UGDH, HK1, NAGK |
| REACTOME_PATHWAY | REACT_216:DNA Repair | 10 | 0.001 | NBN, REV1, POLR2K, POLE, DDB2, LIG3, OGG1, ATM, RAD50, BRCA1 |
| REACTOME_PATHWAY | REACT_152:Cell Cycle, Mitotic | 14 | 0.05 | ITGB3BP, NUP160, BTRC, POLE, ANAPC4, KIF18A, POLA2, MCM8, APITD1, CCND2, PSMA3, CEP290, ANAPC7, NUP43 |
