# Supplementary material for: The small GTPase RhoU lays downstream of JAK/STAT signaling and mediates cell migration in multiple myeloma
Source: Blood Cancer J. 2018 Feb 13;8(2):20. doi: 10.1038/s41408-018-0053-z (PMC5811530; doi:10.1038/s41408-018-0053-z)
Supplement: Supplementary file 4 — Supplementary Figure S1 [file 41408_2018_53_MOESM4_ESM.pptx]

## Slide 1
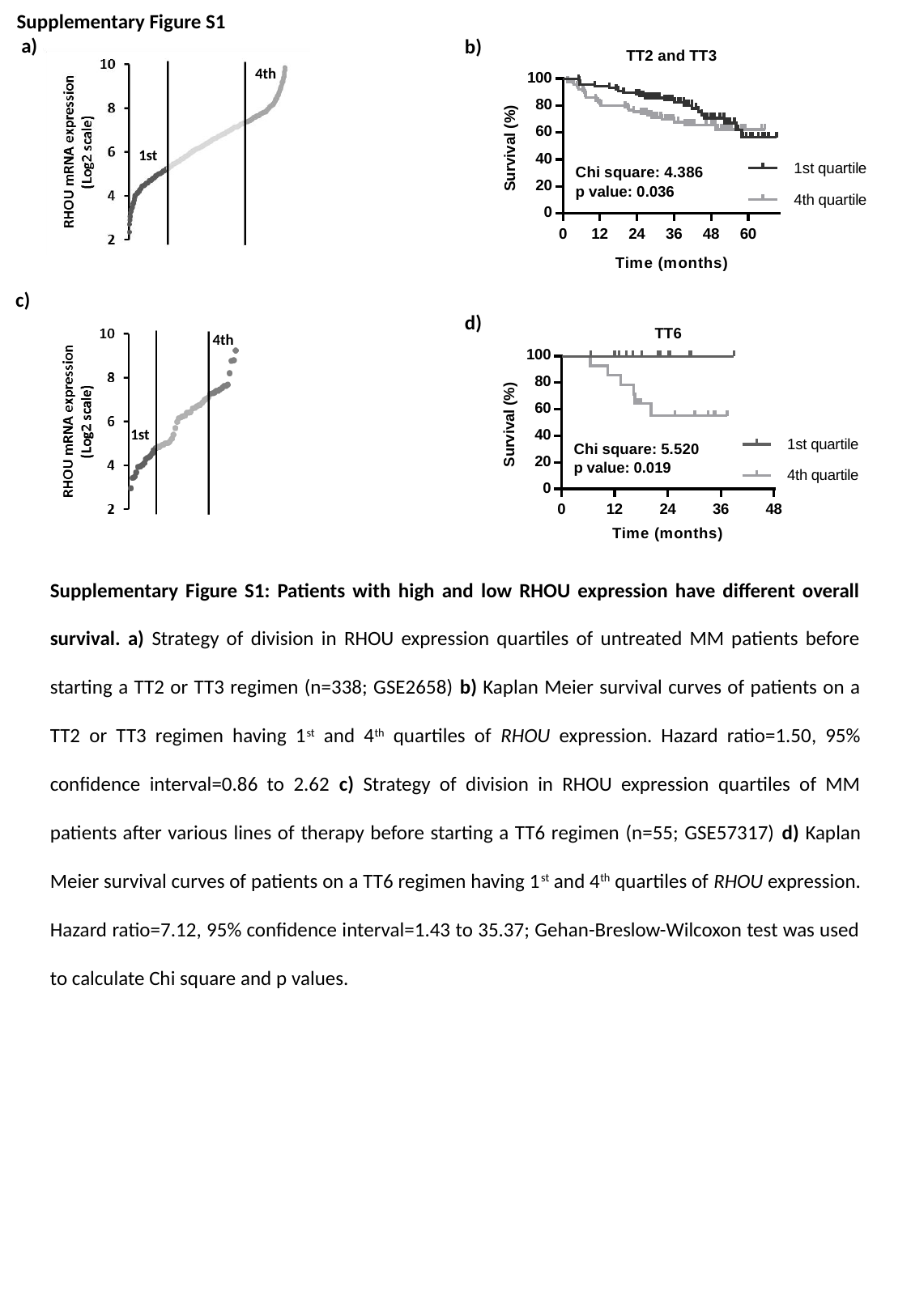

Supplementary Figure S1
 a)
b)
4th
1st
c)
d)
4th
1st
Supplementary Figure S1: Patients with high and low RHOU expression have different overall survival. a) Strategy of division in RHOU expression quartiles of untreated MM patients before starting a TT2 or TT3 regimen (n=338; GSE2658) b) Kaplan Meier survival curves of patients on a TT2 or TT3 regimen having 1st and 4th quartiles of RHOU expression. Hazard ratio=1.50, 95% confidence interval=0.86 to 2.62 c) Strategy of division in RHOU expression quartiles of MM patients after various lines of therapy before starting a TT6 regimen (n=55; GSE57317) d) Kaplan Meier survival curves of patients on a TT6 regimen having 1st and 4th quartiles of RHOU expression. Hazard ratio=7.12, 95% confidence interval=1.43 to 35.37; Gehan-Breslow-Wilcoxon test was used to calculate Chi square and p values.
