# Supplementary material for: The small GTPase RhoU lays downstream of JAK/STAT signaling and mediates cell migration in multiple myeloma
Source: Blood Cancer J. 2018 Feb 13;8(2):20. doi: 10.1038/s41408-018-0053-z (PMC5811530; doi:10.1038/s41408-018-0053-z)
Supplement: Supplementary file 5 — Supplementary Figure S2 [file 41408_2018_53_MOESM5_ESM.pptx]

## Slide 1
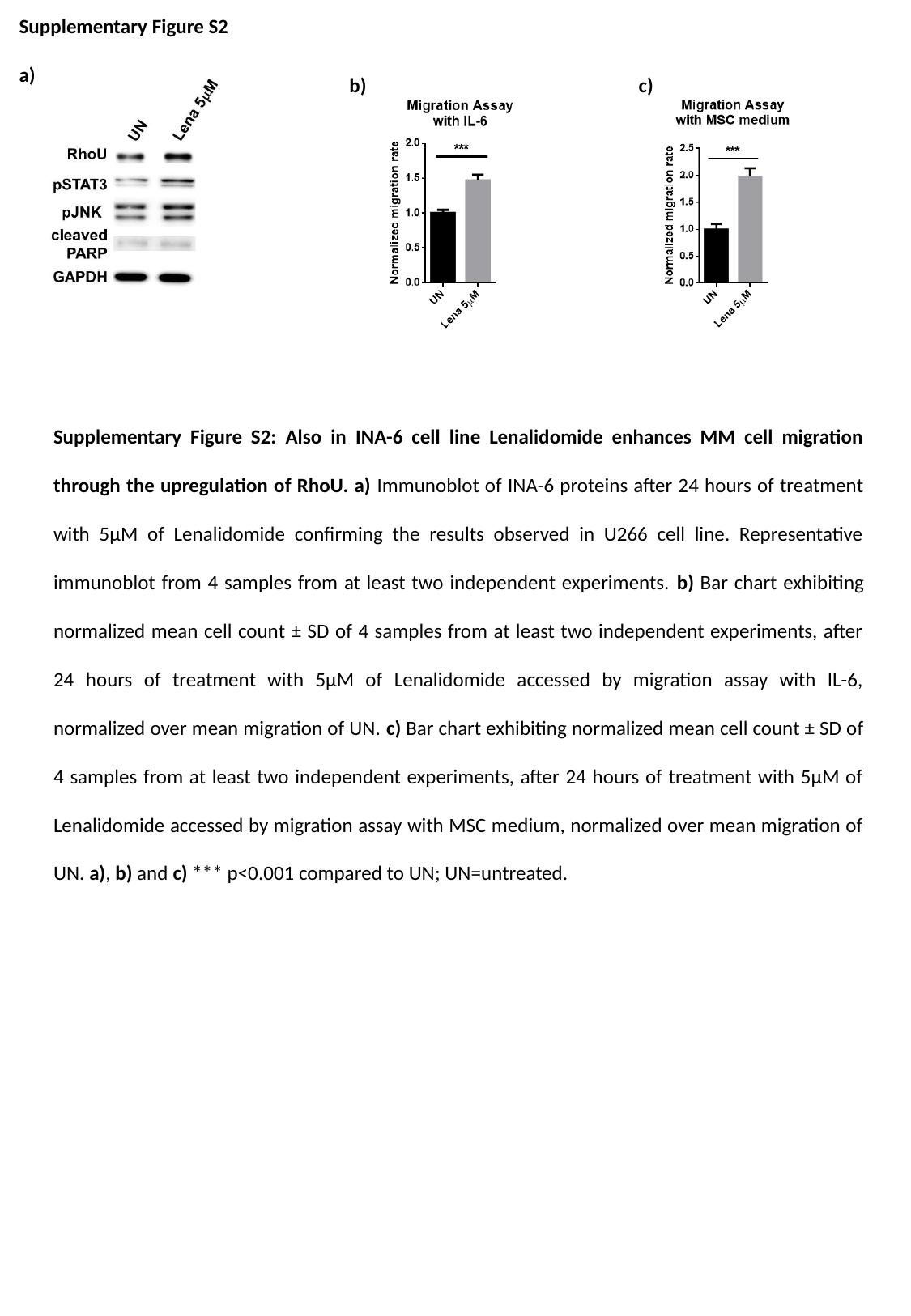

Supplementary Figure S2
a)
b)
c)
Supplementary Figure S2: Also in INA-6 cell line Lenalidomide enhances MM cell migration through the upregulation of RhoU. a) Immunoblot of INA-6 proteins after 24 hours of treatment with 5µM of Lenalidomide confirming the results observed in U266 cell line. Representative immunoblot from 4 samples from at least two independent experiments. b) Bar chart exhibiting normalized mean cell count ± SD of 4 samples from at least two independent experiments, after 24 hours of treatment with 5µM of Lenalidomide accessed by migration assay with IL-6, normalized over mean migration of UN. c) Bar chart exhibiting normalized mean cell count ± SD of 4 samples from at least two independent experiments, after 24 hours of treatment with 5µM of Lenalidomide accessed by migration assay with MSC medium, normalized over mean migration of UN. a), b) and c) *** p<0.001 compared to UN; UN=untreated.
